# Identification of Genetic Variation on the Horse Y Chromosome and the Tracing of Male Founder Lineages in Modern Breeds

**DOI:** 10.1371/journal.pone.0060015

**Published:** 2013-04-03

**Authors:** Barbara Wallner, Claus Vogl, Priyank Shukla, Joerg P. Burgstaller, Thomas Druml, Gottfried Brem

**Affiliations:** Institute of Animal Breeding and Genetics, Department of Biomedical Sciences, University of Veterinary Medicine Vienna, Vienna, Austria; University of Uppsala, Sweden

## Abstract

The paternally inherited Y chromosome displays the population genetic history of males. While modern domestic horses (*Equus caballus*) exhibit abundant diversity within maternally inherited mitochondrial DNA, no significant Y-chromosomal sequence diversity has been detected. We used high throughput sequencing technology to identify the first polymorphic Y-chromosomal markers useful for tracing paternal lines. The nucleotide variability of the modern horse Y chromosome is extremely low, resulting in six haplotypes (HT), all clearly distinct from the Przewalski horse (*E. przewalskii*). The most widespread HT1 is ancestral and the other five haplotypes apparently arose on the background of HT1 by mutation or gene conversion after domestication. Two haplotypes (HT2 and HT3) are widely distributed at high frequencies among modern European horse breeds. Using pedigree information, we trace the distribution of Y-haplotype diversity to particular founders. The mutation leading to HT3 occurred in the germline of the famous English Thoroughbred stallion “Eclipse” or his son or grandson and its prevalence demonstrates the influence of this popular paternal line on modern sport horse breeds. The pervasive introgression of Thoroughbred stallions during the last 200 years to refine autochthonous breeds has strongly affected the distribution of Y-chromosomal variation in modern horse breeds and has led to the replacement of autochthonous Y chromosomes. Only a few northern European breeds bear unique variants at high frequencies or fixed within but not shared among breeds. Our Y-chromosomal data complement the well established mtDNA lineages and document the male side of the genetic history of modern horse breeds and breeding practices.

## Introduction

Mitochondrial DNA (mtDNA) and the paternally transmitted portion of the Y chromosome (NRY) are inherited uniparentally and do not recombine. They accumulate mutations, such as single nucleotide polymorphisms (SNPs), insertions and deletions (Indels) and structural rearrangements [1]–[3]. Genealogies inferred from the distribution of mutations on mtDNA and NRY haplotypes reflect gender-specific population genetic forces. Furthermore, gene conversion i.e. the transfer of a short sequence from the homologous, but non-recombining region on the X- to the Y-chromosome can also contribute to Y-chromosomal variation [4], [5]. mtDNA- and NRY-variant distributions have been widely analysed in humans and in a broad range of wild and domesticated animals to provide indications of the origin of species, domestication processes, the characterization of genetic diversity within and between populations and sex specific demographic behaviors [6]. Depending on the genetic regions analysed, uniparentally inherited markers can be used to trace individual founder lines or families [7]. In livestock, Y-chromosomal and mtDNA variation has been used to study the domestication and population structure of the domestic dog [8]–[10], pig [11], [12], sheep [13] and cattle [14].

Populations of the domestic horse (*E. caballus*) show high levels of mtDNA diversity with limited geographic structure [15]–[19]. Estimates of the coalescence times of mtDNA variants of extant horses far predate the date of domestication [18], [19]. Hence, multiple female lineages contributed to the domestic horse gene pool. Nuclear microsatellite data are also consistent with a scenario of high variation that predates domestication [20].

Nevertheless, genetic variability in the domestic horse represents a paradox: although horses have the largest diversity of maternal mtDNA among domestic species, no noteworthy sequence diversity can be detected on the NRY [21]–[23]. Whereas Y chromosomes of modern horses are clearly distinct from that of the Przewalski horse (*E. przewalskii*) [22] and ancestral Y-chromosomal diversity is found in prehistoric horses [24], the only polymorphism observed in modern horses is a microsatellite mutation found in autochthonous Chinese horse breeds [25]. Screening approaches for the identification of polymorphisms on the domestic horse NRY have proven unsuccessful [21]–[23]. The low NRY diversity in horses is assumed to be the result of the extremely low effective population size of males due to the specific mating behaviour and to several bottlenecks that occurred early in the domestication process [20], [21]. Moreover, Y-chromosomal variation might be further diminished in modern horse breeds by regulated breeding programmes and intensive horse-trading. Today’s European horse breeds (e.g. the Lipizzan horse and the English Thoroughbred) are largely the result of centralized and organized breeding over the past 200 years. The breeding effort has mainly focused on stallions. Breeding programmes have been based on so-called “multiplier studs”, which were founded at the end of the 18^th^ century and were responsible for the production of stallions needed in rural regions. As a result of these early breeding programmes, modern European horse breeds show the consequences of several waves of introgression of imported stallions into local breeds. These include (a) the “Neapolitan” wave from the 15^th^ to the 18^th^ century, when the now extinct “Neapolitan horse” was introgressed; (b) the “Oriental” wave from the late 18^th^ to the late 19^th^ century, when “Original Arabian” stallions, imported from Syria to Egypt, were introgressed and (c) the “English” wave from the early 19^th^ century to the present, when Thoroughbred stallions were introgressed [26]–[28]. In the early 19^th^ century new breeding practices were introduced and rapidly supplanted existing practices. Inbreeding and line breeding concepts became popular and with the integration of private breeding into state breeding programmes the entire population of male horses became highly selected. The result of the modern breeding practices may have been the complete replacement of autochthonous Y-chromosomal variants by imported bloodlines.

In the Lipizzan breed, 89 different sire lines existed in the late 18^th^ century, while only eight are present today [29]. Among these eight male lines, one can be attributed to the “Oriental” wave from the Original Arabian stallion “Siglavy” (born 1810, imported to Lipizza 1814). The other Lipizzan stallion lines derive from the earlier, less documented phase (the “Neapolitan” wave). The male gene pool is even more restricted in the English Thoroughbred, with only three paternal lines remaining [30]. All three lines can be attributed to the “Oriental” wave through the import of the three stallions Byerley Turk, Darley Arabian and Godolphin Arabian. Pedigrees in other European breeds are less well documented but reductions in male lines are similar and an influence of Neapolitan and Original Arabian horses can be observed or is presumed to have occurred. Pedigree information on northern European horse breeds also indicates reduced male diversity. Nevertheless, these breeds, notably the Icelandic horse, might be the only European breeds not to have been subjected to the recent introgression waves [30].

Due to the lack of polymorphic markers on the NRY, it is not possible to trace male-mediated population-genetic dynamics in modern horses. We now describe a systematic screen for horse Y-chromosomal variants in modern domestic horse breeds. As there is only scarce sequence information relating to the horse Y chromosome, we sequenced Y-chromosomal BAC clones to obtain reference sequences for our screen. Based on de novo assembled contigs, we amplified long-range PCR (LRP) products covering about 186 kb for targeted resequencing [31]. To identify variants, we selected (a) nine male horses from phenotypically, genetically and geographically highly distinct breeds, (b) eight Lipizzan stallions, each representing a classical founder line [26], and (c) one Przewalski horse (*E. przewalskii*). LRP products were pooled (“seq-pools”) and sequenced with llluminás Solexa technology. Based on the mutations identified, we describe the first Y-chromosomal haplotypes (HT) in domestic horses and their phylogenetic relationships (a detailed workflow is given in Fig. S1). Furthermore, we present the results of a screen for the distribution of the HTs among purebred modern horse breeds. Using pedigree data, we are able to trace the paternal roots of the extant males. Our data show the strong influence of influential founders, mainly from the Near East and a certain Thoroughbred line, on extant horse breeds.

## Materials and Methods

### Ethics Statement

#### 1) Blood samples

Genomic DNA samples from the breed pool and the Przewalski horse isolated from blood were collected as part of routine diagnostics at the Institute of Animal Breeding and Genetics, University of Veterinary Medicine, Vienna, during the 1970’s, 80′s, and 90′s. For the breed pool, information on breeds was available but the dates of collection, owners and horse identification were not recorded. The Przewalski horse was provided by Dr. Meltzer (Zoo Munich) and the Icelandic horse by B. Wallner.

The Lipizzan horse blood samples were collected before 1999 during an INCO Copernicus project (1996–2001). Permission for the scientific use of the samples was granted by all involved stud farms, which were partners in the project. A summary of the findings was published in “Der Lipizzaner im Spiegel der Wissenschaft” [26], edited by G. Brem, Publisher: Österreichische Akademie der Wissenschaften. We are not aware whether the blood samples were taken during routine diagnostics.

Samples were collected before the establishment in 2004 of the ethics commission of the University of Veterinary Medicine, Vienna.

#### 2) Hair root samples

165 hair samples were recently taken and permission was granted by the private owners or breeding associations. The following breeding associations gave their consent: Associacion française du Lipizzan (France), Escola Portuguesa de Arte Equestre (Portugal), Fundación Real Escuela Andaluza del Arte Ecuestre (Spain), Lipizzanergestüt Piber and the Spanish Riding School (Austria).

450 genomic DNA samples isolated from hair roots derive from routine parentage testing from the archive of the company AGROBIOGEN GmbH, Hilgertshausen. DNA samples kindly provided by Agrobiogen are several years old.

All samples were anonymized.

### 454 Sequencing of BAC Clones

**Figure 1:**
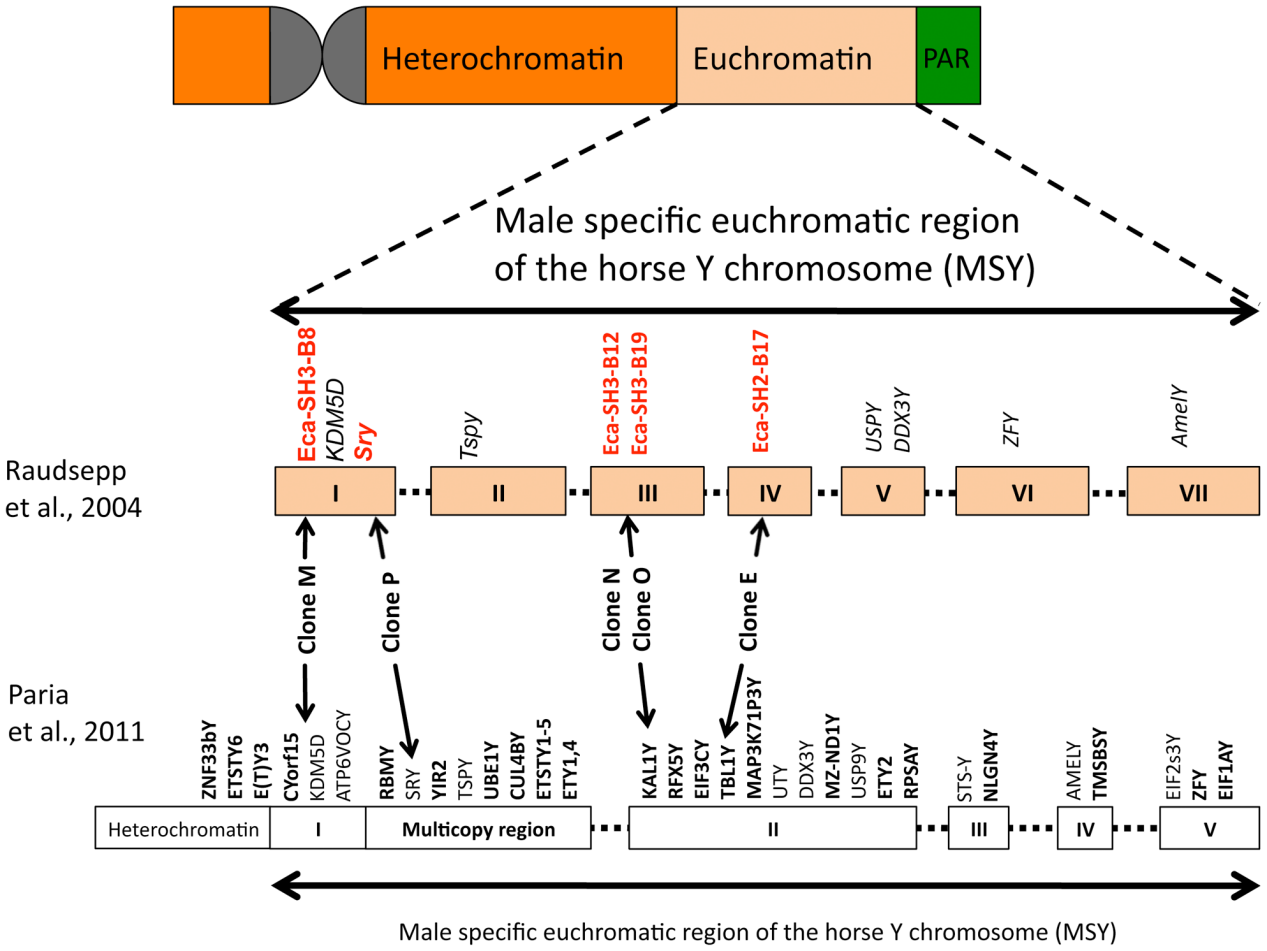
The localisation of the BAC-clones on the horse Y chromosome (ECAY). The heterochromatic, male specific euchromatic (MSY) and the pseudoautosomal region (PAR) of the horse Y chromosome are shown at the top. The seven MSY contigs (I-VII) described in the study of Raudsepp et al., 2004 [33] are illustrated in the middle and indicated as boxes; gaps between contigs are spanned with dotted lines. The locations of some MSY genes are given (italics). The chromosomal position of each BAC clone used in this study is marked by an arrow. On the bottom the approximate positions of the BAC clones on the horse Y-chromosomal gene map after Paria et al. 2011 [40] are indicated.

BAC DNA from 5 Y-chromosomal BAC clones (Fig. 1, Table S1,) [32], [33] was isolated as described at http://dga.jouy.inra.fr/grafra/BAC_DNA_midiprep.htm and treated with RNAseA. BAC DNA was purified with the Mammalian Genomic DNA Miniprep Kit (Sigma-Aldrich, cat. no G1N350-1KT). Parallel sequencing on the 454 Roche system was performed according to standard procedures at the Core Facility Molekularbiologie (Meduni Graz, AT). 50 ng of total BAC DNA were prepared with the Nextera™ DNA Sample Prep Kit (Epicentre, cat.no. NT09115) according to the manufacturer’s instructions. DNA was incubated in the enzyme buffer for 5 minutes at 55°C and purified with the Qiagen MinElute PCR Purification Kit (Qiagen cat.no. 28004) and titanium adaptors were ligated to the fragments. After emulsion PCR (GS titanium emPCR reagents Lib-L Roche cat. no. 05618444001), bead recovery and enrichment sequencing was performed with the GS Titanium Sequencing Reagent XLR70 (Roche, cat. no. 05233526001) according to Roche standard 454 protocols.

The raw sequence reads were filtered and trimmed to reject too short or bad quality sequences. No ambiguous base was allowed and the minimum sequence length exceeded 50 bases. Separate assemblies were performed for BAC clones M, P and E using Roche GS De Novo Assembler v2.3 to generate the contigs. Overlapping clones N and O were assembled together. Contig sequences were trimmed for BAC vector sequence and *E.coli* genome sequences. Assembled sequences longer than 5000 bp were selected for further analysis (see Table S2 for details).

### Analysis of Contig Sequences

Contig consensus sequences generated in each library were screened for homologies by manual blast search (blastN) against the complete nucleotide collection in Genbank and by BLAT (http://genome.ucsc.edu/cgi-bin/hgBlat) against the horse genome. Repetitive elements were identified with Repeat Masker (http://www.repeatmasker.org/).

### Long–range PCRs

Primers were designed with Primer3. Amplicon length ranged from 5.5 to 11.8 kilobase pairs (kb) and some contigs overlapped (primer sequences and amplicon length are listed in Table S3). PCR products were amplified with the Expand Long Template PCR System (Roche, cat. no. 11681842001) as described in the Kit and checked for male specificity by comparative amplification of blood genomic DNA from male and female horses (Fig. S2). Sequence specificity was controlled by restriction enzyme digests.

### DNA Amplification for Sequence Analysis

To maximize diversity in the sample set, nine male purebred domestic horses from phenotypically different breeds and geographically distinct regions were selected for the first seq-pool (“breed”). The second pool (“lipp”) contained 8 Lipizzan stallions, each representing a particular paternal founder line [26]. In the third pool (“prz”), only one Przewalski horse was sequenced (Table S4). To detect all variants in a pooled sample [31], single LRP products were generated from each individual using high molecular-weight genomic DNA isolated from whole blood. The LRP products were visualized on a 0.8% agarose gel and the concentration of each product was measured with the Qubit ds DNA HS Assay (Invitrogen cat. no. Q32851). In case of multiple PCR products the Y chromosome specific amplicon was isolated from the agarosegel prior NGS library preparation. Y-specific LRP products were pooled equimolarily and cleaned with the High Pure PCR Product Purification Kit (Roche, cat. no. 11732668001), resulting in 2 µg clean LRP product per pool.

### Illumina Library Preparation and Data Generation

The pools were fractionated to a size range of 300–700 bp using a Covaris sonicator and fragments were purified using the High Pure PCR Product Purification Kit. For high-throughput sequencing, libraries were prepared with NEBNext DNA Library Prep (NEB, cat. no. E6000S) using NEBNext Multiplex Oligos (NEB cat. no. E7335L) to index the three pools for multiplex sequencing according to the manufacturer’s instructions. Indexed pools were submitted together on one lane of 76 bp paired-end sequencing at the CSF NGS Unit (http://csf.ac.at/) using the Illumina GA II system [34]. The quality of the data was checked with FASTQC [35], sequences were aligned to the reference BAC sequence with BWA [36] and quality control (filtering) was performed with SAMtools [37]. After quality control and removal of duplicate reads, ∼4500×, ∼5000× and ∼2500× mapped sequence coverage of the available Y reference sequence was obtained from Lipizzan, domestic and Przewalski samples, respectively. SNP calling (identifying positions that differed from the reference sequence) was performed with SAMtools and in-house python scripts.

### Data Filtering to Identify Candidate Mutations

As there were nine individuals in the pool “breed”, theoretically 11.1% of genomic DNA was contributed by each horse in the sequencing sample. Considering random errors and experimental bias at the various stages of library preparation, 8% was decided as a safe threshold for SNP calling. As only one individual was sequenced in the prz-pool, sites that differed from the reference in the same proportion in all three pools were assumed to be base-calling or alignment errors. For the second-class SNPs, we lowered the threshold for SNP calling to 6% minor allele frequency and/or allowed 3% in the prz-pool horse.

### Verification of Candidate Polymorphic Sites by Sanger Sequencing and Sequence Analysis

For the filtered candidate mutations, we designed PCR primers by using Primer3 to amplify 240–760 bp fragments (primer information in Table S5). PCR products were amplified from genomic DNA from each horse from the breed-, lipp-, and prz-pools and from a second Shetland pony (n = 18). Amplicons were resequenced by conventional Sanger sequencing for forward and reverse strands at LGC genomics. See Figs. S3, S4, S5, S6 and S7 for the sequence alignment with primer binding sites. PCR products amplified from male and female DNA and the results of the Sanger sequencing for each polymorphic region. *E. przewalskii*-determining sites (positions 4086 and 4161 on contig YM23) and Eca-Y2B17 [22] and intron2 from AMELY [24], representing regions harbouring ancestral diversity, were sequenced for all horses in the pools. Sequences were aligned with the CLC workbench. Nucleotide diversity was calculated in R according to the formula of Nei [38].

### Microsatellite Analysis

Individuals were characterized using five equine Y-chromosomal microsatellites described previously [23] (modification and results are given in Table S6). Genotypes were determined with MegaBACE 500 at the University of Veterinary Medicine, Vienna. Electropherograms were evaluated using MegaBACE Genetic Profiler v2.2 (GE Healthcare). Microsatellite variation was investigated for 100 domestic horses from various breeds (Table S7), including the horses that were used for the seq-pools and three Przewalski horses.

### Screening of Domestic Horses for Y Haplotypes

615 male horses, representing 58 mainly European horse breeds, were screened for their Y-chromosomal haplotypes. Genomic DNA samples isolated from hair roots (purified with nexttec™) derive from routine parentage testing (for details see Table S7). Genotyping was performed using the Sequenom MassARRAY iPLEX system (Sequenom, Germany) at the Department for Agrobiotechnology, IFA Tulln. A section of DNA containing the variant position was amplified from each individual by PCR, before a high-fidelity single-base primer extension reaction over the SNP being assayed was undertaken, using nucleotides of modified mass. The different alleles therefore produce oligonucleotides with mass differences that can be detected using highly accurate Matrix-Assisted Laser Desorption/Ionization Time-Of-Flight mass spectrometry [39]. The five SNP multiplex assay was designed using the Sequenom Assay Design 3.1 software, PCR primers and extension primers shown in Table S8. SNP genotyping was performed using the iPLEX® GOLD Complete Genotyping kit with SpectroCHIPs® II in the 384 format (Sequenom, Germany) in duplicate and female genomic DNA was included as a negative control. We followed the manufacturer’s protocol with a single modification: to reduce unspecific primer extension, 5 ng sheared salmon sperm DNA (Invitrogen, Austria) per reaction was added to the PCR mastermix. Results were analysed with the Sequenom Typer 4.0 software (Sequenom, Germany) and the results validated by SANGER resequencing of ten (when available) alleles from each position.

The screening criteria for Shetland pony HT6 were (a) no results in Y-E17.1mut_SNP1 & SNP2, and (b) amplification of the 966 bp deletion and visualization on an Agarose gel (Fig. S8, Table S8).

### Pedigree Analysis

For pedigree analysis, web-based databases were used: for Thoroughbreds the Pedigree Online Thoroughbred Database (availiable at: www.pedigreequery.com/) and the Galoppsieger database (www.galopp-sieger.de/); for Shagya Arabians the Shagya Database (www.shagya-database.ch/hengste.php); for Icelandic horses the Stormhestar Database (www.stormhestar.de/german/default.asp) and for many other breeds the Pedigree Online All Breed Database (www.allbreedpedigree.com) or the Sporthorse Horse Show and Breed Database (www.sporthorse-data.com/breed.htm). All database informations were last accessed in October 2012.

Thirteen breeds (Appaloosa, Barb, Konik, Mangalarga Paulista, Mangalarga Marchador, New Forest, Paint, Russian Arabian, Saddlebred, Shire, Tinker horse, Wuerttemberg, Camargue) are not included in the pedigree analysis, as we had no males with a proven ancestry. For founder classification we call Arabian, so-called Oriental and Turkoman horses (i.e. horses from the middle East ranging from Turkmenistan to Egypt) that were imported to Europe in the 18^th^ and 19^th^ century “Original Arabians”.

## Results

### Identification of Y Chromosome Polymorphisms

To detect polymorphisms, we generated reference sequences from the horse Y chromosome by 454 sequencing of 5 Y-chromosomal BACs from certain regions on the chromosome (Fig. 1, Table S1,) [23], [32], [33], [40]. Sequence reads were de novo assembled and 11 contigs and an *Sry* containing BAC sequence (AC215855.2) were selected for designing 21 LRPs, covering a total of 186,122 bp (Table S3). Y-chromosomal specificity was checked by comparative amplification of the LRPs on male and female DNA (Fig. S2).

We produced Y-chromosomal LRP products from 17 domestic and one Przwalski horse separately and pooled the products (Table S4). LRPs of nine males from different breeds (pool-breed), of eight Lipizzan stallions (pool-lipp) and the Przewalski horse (pool-prz) were sequenced with the Illumina platform to high depth. Whereas the Przewalski horse showed 37 base substitutions and one 3051 bp deletion compared to the domestic horse samples, only 4 positions/regions promised to be polymorphic within the domestic horse pools. With relaxed criteria (see Materials and methods) another 19 second-class SNPs were added. For validation, we amplified the region spanning each polymorphic candidate from each domestic horse in the seq-pools and sequenced them by conventional Sanger sequencing. The four top candidate regions were confirmed but none of the 19 second-class SNPs were (Table S5). The resequencing results and the male specificity of the confirmed candidates are shown in Figs. S3, S4, S5, S6 and S7. The variants detected in domestic horses were two SNPs, one single base deletion and a complex variant consisting of 20 SNPs and four indels. In addition we identified a 966 bp deletion in a Shetland pony and a transition in a second Przewalski horse during the screening procedures (Table S9, S10, Fig. S1, Fig. S8).

The two SNPs were found on discrete contigs (YE17 and YXX_24I23) but the indel polymorphism, the complex mutation and the 966 bp deletion are all located in close proximity or even overlapping in a restricted region on contig YE3. Screening for homologies to this region in the horse genome using BLAT revealed high similarities (>95%) with the homologous region on the horse X chromosome and the occurrence of a LINE element approximately 700 bp upstream of the region that is mutated in the domestic horse (Fig. S9). The alignment of X - and Y-sequences shows the need for careful selection of PCR amplification primers to amplify Y-chromosomal sequences. Furthermore, one can see from the alignment (Fig. S5) that the multiple variants on locus YE3 - Pos 1007–12040 conform to the X- rather than to the Y chromosome for a length of 300 bases. We thus assume that the complex variant on YE3 - Pos 1007–12040 comprising 25 SNPs and four indels is the result of a gene conversion event between the X and Y chromosome [5]. The deletion of a single T on locus YE3 - Pos 10594 is also found on the homologous X-chromosomal sequence (Fig. S4) and thus another putative gene conversion event.

### Y-chromosomal Diversity in Modern Horses - Haplotype Network and Estimation of Divergence Time

**Figure 2:**
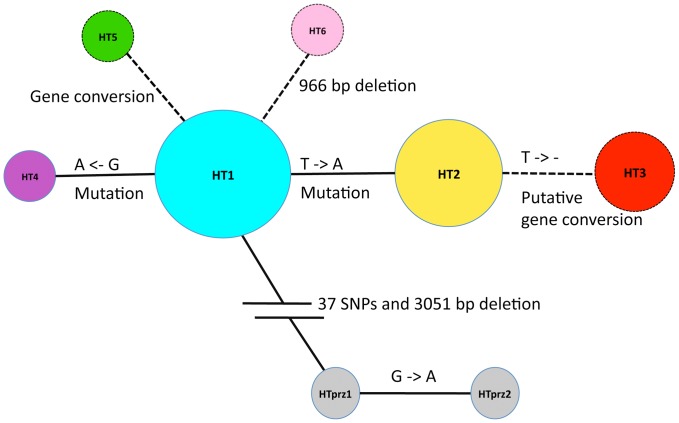
Haplotype network of the six modern and two Przewalski horse HTs. Circles represent the haplotypes with the area proportional to the observed frequency in 20 male horses in the initial Y-chromosomal sequence analysis (Table S4). HT1, n = 7 (three Lipizzan, two Arabian, one Shetland pony, one Shire horse); HT2, n = 5 (five Lipizzan); HT3, n = 3 (one Thoroughbred, one Trakehner, one Quarter horse); HT 4 (one Icelandic horse), HT5 (one Norwegian Fjord horse), HT6 (one Shetland pony), HTPrz1 (one Przewalski horse), HTPrz2 (one Przewalski horse). A dashed line between the haplotypes indicates, that the polymorphism is located on the highly variable contig YE3, which was omitted when estimating divergence time and nucleotide diversity.

The polymorphisms result in six haplotypes in the domestic and two in the Przewalski horse (Tables S9, S10 and S11). The ancestral and the derived status in modern horses were determined by comparison to the Przewalski horse sequence. The haplotype network in Fig. 2 gives the relationship between the HTs. *E. caballus* haplotypes are separated by only one mutational or gene conversion step, i.e. there is no deep bifurcation with many segregating mutations. Three haplotypes (HT1-3) occur at relatively high frequencies. HT1 is the ancestral haplotype when rooted with the Przewalski Y haplotype. HT2 is differentiated from HT1 by a single nucleotide mutation. The deletion that defines HT3 arose on the background of HT2. Clearly, HT1 is the most prominent haplotype in the evolution of modern horse breeds: of the five mutations observed in the panel, four arose on the background of HT1 and only one on the background of HT2. Thus, although the proportion of HT1 and HT2 in our sample is about equal, all except one variant arose on the background of the older and thus evolutionarily more important HT1. Lippold et al. found a Y-chromosomal HT in an ancient domestic horse that differed at three positions from the common domestic horse HT chromosome [24]. We observed none of these ancient bases when screening a set of extant horse breeds that represent all six haplotypes. The Y chromosome of the Przewalski horse forms a separate clade with a sequence divergence of 0.021% from *E. caballus*.

For inference of population genetic diversity and divergence time estimates, we rely only on putative single nucleotide polymorphisms, i.e., we exclude the region affected by gene conversion (YE3). The rate of gene conversion is known to depend strongly on the genomic location, and is particularly high at translocation hotspots [41], [42]. The prediction of these hotspots seems complex [4], but the occurrence of a LINE element and the X/Y-sequence similarity indicate that the YE3 region is a candidate. Furthermore, we infer from pedigree data, that the deletion on YE3 leading to HT3 arose, presumably by a gene conversion event, very recently (see below). In addition, the occurrence of an independent 966 bp deletion in HT6 points to a general instability of the YE3 region. We thus base our estimates of diversity and dating solely on the observation of two SNPs (YXX_24I23 - Pos 25345 and YE17.1 - Pos 1277) per 170 731 bp of total length. We assume a rate of single nucleotide mutations per generation of about 1.1–5×10^−8^ as found for humans [1], [43], [44] and expect the rate of mutations per generation in our dataset to be: µ× (sequence length) ≈1,8 to 8×10^−3^. Thus only 234 to 1064 meioses are theoretically necessary to give rise to the two SNPs over the region under investigation. We conclude that the present Y-chromosomal diversity in modern breeds most likely arose by mutations from HT1 after domestication, about 6000 years or 1000 generations ago [45]. We note, however, that the NRY is inherited as a single locus, so inferences based on the NRY are subject to large stochastic errors for parameters such as the overall tree depth.

Considering the frequencies of haplotypes with SNPs, pairwise nucleotide diversity [38] is π = 3.71×10^−6^, an extremely low value compared to those observed in domestic pigs (π = 1.38×10^−3^) [11], [12] and dogs (π = 2.91×10^−4^ *)* [46].

### Phylogeography of Horse Y-chromosomal Haplotypes

**Figure 3:**
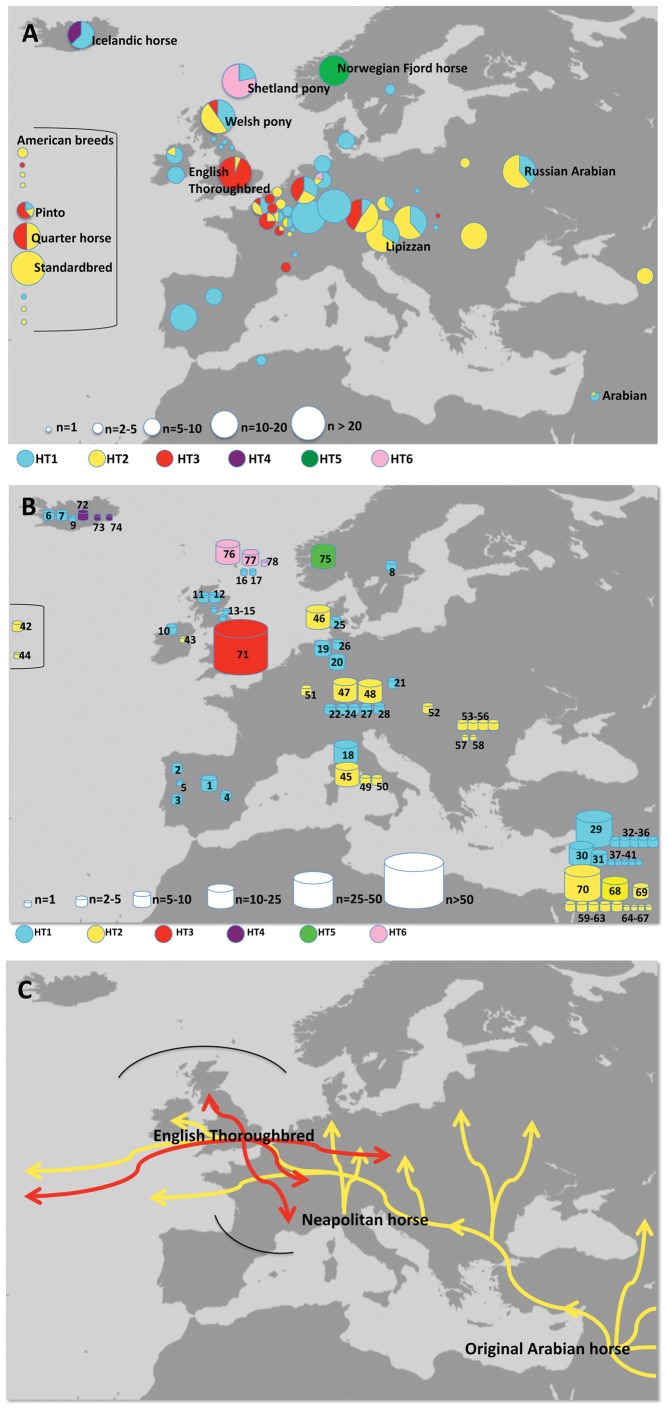
Geographic distribution and history of Y-haplotypes in modern horse breeds. (a) Geographic distribution of Y-chromosomal haplotypes in a set of modern horse breeds. Only a few important breeds are specified, the full list with information on breeds and HT frequencies is given in Table S7. (b) Origin of modern domestic horse founders deduced from pedigree data. Each founder is represented by a drum with its size proportionally to the number of offspring in the dataset. The number in the drums serve as founder identifiers. Detailed information on founders (name, year of birth, breed, origin, information on import) is listed in Table 1. (c) Male introgression routes deduced from the pedigree and the distribution of HT2 and HT3 in our dataset. HT2 (yellow arrows) arrived from South-East at early times and has been spread during the Neapolitan and Oriental introgression waves, but did not reach Northern Europe and the Iberian peninsula. The English wave in red is well documented through pedigree data and the spread of HT3 (red arrows). Due to the ubiquitous occurrence of HT1, this haplotype is not considered. The black solid lines reflect the limits of the observation of HT2 and HT3.

We examined the distribution of the Y-chromosomal haplotypes in 615 males from 56, mainly European, domestic horse breeds. To avoid father-son pairs or paternal brothers in the dataset, only purebred horses with confirmed paternity were investigated. The results are listed in Table S7. 91% of the male horses were found to carry one of the three major haplotypes. The ancestral HT1 is distributed across almost all breeds and the entire geographical region under investigation, underlining its importance (Fig. 3A). HT2 is also found at high frequencies across a broad range of breeds, although not in the northern European breeds and not in horses from the Iberian Peninsula. HT3 is almost fixed in the English Thoroughbred and is further distributed across many warm-blooded breeds. HT4-6 are only found in three local northern European breeds but in high frequencies in these breeds: HT4 in half the Icelandic horses and HT6 in 74% of the Shetland ponies while HT5 is fixed in Norwegian Fjord horses. We undertook genotyping of five Y-specific microsatellite markers [23] from a subset of 100 horses distributed over all HTs. Microsatellite analysis showed a uniform pattern of amplification over all domestic horses (Table S6), distinct from the Przewalski horse at one locus, as in previous studies [23], [25].

### Incorporation of Pedigree Data to Uncover Founder Traces

We used the documentation of horse ancestry in studbooks and pedigree data from databases to trace the origin of the purebred horses. Sufficient pedigree information was available for 418 (67.97%) males from our dataset. Whereas the paternal ancestry is accurately documented back to the 18^th^ century for old historic breeds such as the English Thoroughbred and the Lipizzan horse, data on most other modern breeds only extend to about 1900. Pedigree analysis revealed that the 418 males from 43 breeds originate from 78 paternal founders whose geographical/breed origin is shown in Fig. 3B. Founder specifications and haplotype information on renowned stallions are available in Table 1. No founder from the Iberian Peninsula, the British Isles (with the exception of a Connemara pony born in 1921, whose origin and possible Arabian influence is unclear) or northern Europe shows HT2. Instead, HT1 predominates, although autochthonous haplotypes (HT4, 5 and 6) are present at high frequencies in local breeds or are even fixed (e.g. in the Norwegian Fjord horse). In contrast, central, eastern and southern European as well as Original Arabian founders from the near East exclusively show HT1 and HT2 in about equal proportions.

**Table 1:** Founders contributing to modern horses.

Founder identifier (Fig. 3B)	Founder name	Born	Inferred haplotype	Breed and/or geographical origin	Documented import	Descendants in dataset (n)	Modern breeds distributed
1	Favorito	1889	HT1	Spanish purebred/Spain		6	Pura Raza Espaniola
2	Perola	1917	HT1	Lusitano/Portugal		4	Lusitano
3	Nice	1915	HT1	Lusitano/Portugal		3	Lusitano
4	Descindido	1840	HT1	Spanish purebred/Spain		2	Pura Raza Espaniola
5	Marabo	1905	HT1	Alter Real/Portugal		1	Lusitano
6	Brúnn frá Svaðastöðum	1900	HT1	Icelandic horse		5	Icelandic horse
7	Brúnn frá Árnanesi	1910	HT1	Icelandic horse		4	Icelandic horse
8	Hingst fr Gubrandsdalen	1846	HT1	Dole Gudbrandsdal/Norway		2	Swedish coldblood trotter
9	Kjarval frá Sauðárkróki	1981	HT1	Icelandic horse		1	Icelandic horse
10	Mountain Lad	1928	HT1	Connemara/Ireland		3	Connemara
11	Defence	1896	HT1	Welsh/UK		3	Connemara pony
12	Charlie	1880	HT1	Welshmountain/UK		3	Riding Pony, Dartmoor Pony
13	Trotting Comet	1836	HT1	Dale or Exmoor/UK		1	Fell Pony
14	Broccoli	1855	HT1	Welsh/UK		1	Welsh-D
15	Prince Llewelly	1904	HT1	Welsh/UK		1	Connemara pony
16	Fairy Prince	1940	HT1	Shetlandpony/UK		1	Tigerpony
17	Pallieter de Bevelbeekhof	1960	HT1	Shetlandpony/UK		1	Shetland pony
18	Conversano	1767	HT1	Neapolitan Horse/Italy		12	Lipizzan horse
19	Nemo	1885	HT1	Friesian/Netherlands		8	Baroque Pinto, Friesian, Kladruby
20	Old Flyer	1830	HT1	Brandenburg/Germany		8	Welsh-B, Welsh-D
21	Sacramoso Olomouc	1800	HT1	Kladruber/Czech Republik		4	Kladruby
22	Amor	1888	HT1	Pinzgauer horse/Austria		4	Noriker
23	Bravo 149	1877	HT1	Pinzgauer horse/Austria		4	Noriker
24	635 Vulkan	1887	HT1	Pinzgauer horse/Austria		4	Noriker
25	Zarif Sejer	1921	HT1	Fredriksborger/Denmark		3	Knabstrupper
26	Smoky	1955	HT1	Spotted horse/Denmark		3	Knabstrupper
27	80 Arnulf 55	1866	HT1	Pinzgauer horse/Austria		3	Noriker
28	126 Optimus	1890	HT1	Pinzgauer horse/Austria		3	Noriker
29	El Bedavi	1830	HT1	Arabian	1833, Babolna	34	Haflinger
30	Siglavy	1810	HT1	Arabian	1814, Lipizza	11	Lipizzan horse
31	Kuhailan Haifi	1923	HT1	Arabian, desert bred	1931, Poland	6	Shagya Arabian, Arabian, Partbred Arabian, Riding pony
32	Ibrahim	1899	HT1	Arabian/Egypt	Poland/1910 GB	3	Riding pony, Welsh-A, Arabian
33	Khalil a Saklawi Jidran	1876	HT1	Arabian/Egypt	1910, GB, 1925 BRD	3	Arabian, AngloArabian, Shagya Arabian
34	Koheilan Adjuze	1876	HT1	Arabian, desert bred	1885 Babolna	2	Shagya Arabian
35	Kuhailan Zaid	1924	HT1	Arabian, desert bred	1931, Babolna	2	Shagya Arabian
36	Siglavy Bagdady	1895	HT1	Arabian, desert bred	1902, Babolna	2	Shagya Arabian
37	Dahoman	1846	HT1	Arabia, Syria	1852, Babolna	1	Shagya Arabian
38	Hadban	1891	HT1	Arabian, Egypt	1897, Babolna	1	Shagya Arabian
39	Ilderim	1894	HT1	Arabian	1901, Poland	1	Austrian Warmblood
40	Mahmoud Mirza	1851	HT1	Arabian, Iraq	1866, Babolna	1	Shagya Arabian
41	Barq	1840	HT1	Arabian/Egypt	1880, GB	1	Riding pony
42	Traveler	1880	HT2	Quarter horse/U.S.		3	Quarter horse
43	Connemara Boy	1921	HT2	Connemara pony/Ireland		1	Connemara pony
44	Mahoma I	1900	HT2	Paso Fino/Colombia		1	Paso Fino
45	Neapolitano	1790	HT2	Neapolitan Horse/Italy		13	Lipizzan horse
46	Pluto	1765	HT2	Fredriksborger/Denmark		12	Lipizzan horse
47	Favory	1779	HT2	Kladruber/Czech Republic		10	Lipizzan horse
48	Maestoso	1773	HT2	Kladruber/Czech Republic		9	Lipizzan horse
49	Pepoli	1767	HT2	Orig. Italian/Italy		4	Kladruby
50	Tulipan	1880	HT2	Neapolitan Horse/Italy		3	Lipizzan horse
51	Trinket 1883	1883	HT2	German classic pony/Germany		2	German Shetland pony
52	Incitato	1802	HT2	Siebenbuerger stallion/Hungary		2	Lipizzan horse
53	Goral	1898	HT2	Hucul/Romania		5	Hucul
54	Hroby	1894	HT2	Hucul/Romania		3	Hucul
55	Ousor	1929	HT2	Hucul/Romania		2	Hucul
56	Prislop	1932	HT2	Hucul/Romania		2	Hucul
57	Gurgul	1924	HT2	Hucul/Romania		1	Hucul
58	Polan	1929	HT2	Hucul/Romania		1	Hucul
59	Gazlan	1840	HT2	Arabian, desert bred	1852, Lipizza	4	Shagya Arabian
60	O Bajan	1881	HT2	Arabian	1886, Babolna	3	Shagya Arabian, Austrian Warmblood
61	Shagya	1830	HT2	Arabian	1836, Babolna	3	Shagya Arabian
62	Bairactar	1813	HT2	Arabian, desert bred	1817, Weil	2	Riding pony
63	Geok Pischik	1890	HT2	Arabian	1895, BRD	2	Ahkal-Theke
64	Dahman Amir	1887	HT2	Arabian	1890 Szamrajówka	1	Partbred Arabian
65	Latif	1903	HT2	Arabian	1909, Pompadour	1	Partbred Arabian
66	Mersuch	1898	HT2	Arabian	1902, Babolna	1	Partbred Arabian
67	Souakim	1894	HT2	Arabian	1899, Weil	1	Shagya Arabian
68	Byerley Turk	1684	HT2	Arabian	1689, England	8	Warmblood, English Thoroughbred, Hannoveran, Oldenburg, Quarter Horse, Sachsen Anhaltiner
69	Godolphin Arabian	1724	HT2	Arabian or Turkoman	1729, England	5	Pinto, Austrian Warmblood, Warmblood, Hungarian Warmblood
70	Darley Arabian, exluding descendants of Whalebone	1849	HT2	Arabian, Syria	1704, England	44	Standardbred, Oldenburger, Trakehner, Warmblood, Dutch Warmblood, Welsh-A, Welsh-B
71	Darley Arabian, via Whalebone	1807	HT3	Arabian, Syria		59	Bavarian Warmblood, Austrian Warmblood, English Thoroughbred, Hanoverian, Holstein, Oldenburg, Quarterhorse, Partbred Arabian, Riding pony, Rhinelander Horse
72	Hárekur frá Geitaskarði	1915	HT4	Icelandic horse/Iceland		5	Icelandic horse
73	Blettur frá Vilmundarstöðum	1946	HT4	Icelandic horse/Iceland		1	Icelandic horse
74	Ísleifs-Gráni frá Geitaskarði	1910	HT4	Icelandic horse/Iceland		1	Icelandic horse
75	Njal	1891	HT5	Norwegian Fjord/Norway		15	Norwegian Fjord horse
76	Jack	1817	HT6	Shetlandpony/U.K.		14	Shetland pony
77	John Bain	1880	HT6	Shetlandpony/U.K.		7	Shetland pony, Tigerpony
78	Prince of Thule	1872	HT6	Shetlandpony/U.K.		1	Shetland pony

Stallions with descendants in our dataset are listed, giving their origin, HT and their distribution in extant horse breeds (as estimated from our dataset).

At least 202 (48.3%) of the modern horses in our dataset obtained their Y chromosomes from Original Arabian founders. Among the HT2 ancestors are many influential stallions, including the famous founders of the English Thoroughbred: Darley Arabian, Byerley Turk and Godolphin Arabian.

**Figure 4:**
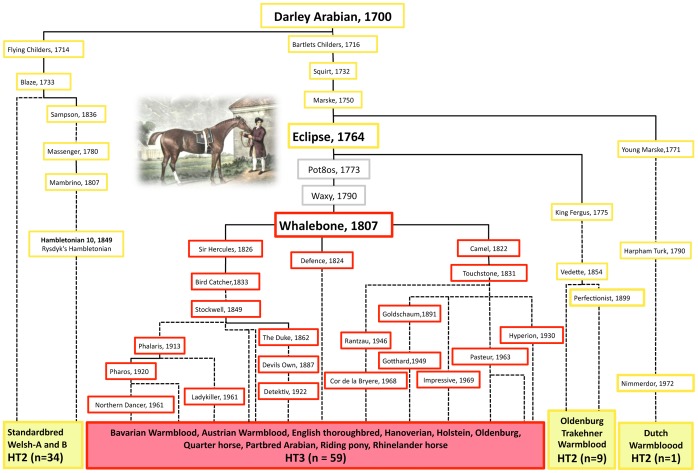
Pedigree of Darley Arabians progeny depicting the origin of HT3 from HT2. Breeds of analysed males are listed on the bottom and the haplotypes of their ancestors are reconstructed (HT2-yellow, HT3-red, unknown-grey). Selected famous stallions are shown by name; dotted lines connect relatives where at least one ancestor is omitted. No descendants from “Pot8os” and “Waxy” were available apart from “Whalebone, 1807”. The mutation leading to HT3 must have occurred either in the germline of stallion “Eclipse” [54] or in his son “Pot8os” or in his grandson “Waxy” and rose to very high frequency in the English Thoroughbred and many sport horse breeds through the progeny of the stallion “Whalebone”.

The English Thoroughbred, which is best known for its use in horse racing, has a complete studbook since 1791 and all registered males can be traced back to one of three popular founders. Among them, the paternal line of Darley Arabian currently represents almost all male Thoroughbreds [47]. Pedigree analysis revealed that all HT3-carrying males can be traced to the single Thoroughbred “Whalebone”, born 1807” a son of “Pot8os”. Hence, the mutation leading from HT2 to HT3 must have occurred either in the germline of the famous racehorse “Eclipse” or in that of his son “Waxy” or grandson “Pot8os” (Fig. 4). The frequency of HT3 rose to 96.5% in the English Thoroughbred and to 41% in modern sport horse breeds in our dataset within 15–20 generations (Fig. 3A). Among 418 long pedigrees, we observed no obvious pedigree errors in the English Thoroughbred and Standardbreds, whereas 17 (4.06%) errors were found in Shetland ponies and Lipizzan, Warmblood and Welsh horses. This observation does not permit us to check the correctness of the studbook, which must be undertaken with maternal lines using mtDNA [48]–[51]. The low initial variation in the founders leads to a low resolution, making pedigree errors in the male line difficult to detect.

## Discussion

The analysis of Y-chromosomal and mtDNA markers offers an invaluable tool for the demographic characterization of populations. To date, the domestic horse was the only livestock species for which paternal lines could not be traced due to the lack of Y-chromosomal variability. We have identified five variants that presumably arose independently and that result in three major and three breed-specific HTs.

Of these five variants, only two arose by single basepair mutations. The other three events arose in a restricted region YE3 with extensive sequence similarity to a X-chromosomal region and consist of a gene conversion tract of about 300 basepairs, a single basepair deletion, which may also be caused by a gene conversion, and a deletion of about 900 bps. X to Y gene conversion strongly influences allelic diversity in specific human Y-chromosomal regions [4], [41]. In this study, we report the first observation of an X to Y gene conversion in a farm animal. The hyperpolymorphic region YE3 may be a hotspot for gene conversions and structural rearrangements on the horse Y chromosome, but a closer investigation is needed.

All domestic horse HTs are closely related. HT1, which is the ancestral haplotype as inferred by comparison with the Przewalski horse, seems to be the only haplotype to have survived through domestication to extant breeds. All other HTs arose directly or indirectly from HT1, presumably after domestication. The finding of low nucleotide diversity of the modern horse Y chromosome is consistent with previous studies, in which no variation was detected [21]–[23]. As the establishment of haplotypes depends on the individuals selected for the initial screen the significance of ascertainment bias has to be kept in mind [6], [52]. To overcome this problem, we screened microsatellite markers in a random sample over all haplotypes/regions and detected no variation. As microsatellites are highly mutable, the absence of significant microsatellite variation on the horse Y chromosome confirms the very recent origin of all haplotypes in our sample. Since we only used puredbred horses from a restricted region we note, that the global horse population may harbour more y-chromosomal variability.

The low diversity of the Y chromosome contrasts with the high diversity of mtDNA haplotypes observed in modern horses [15]–[19]. The difference is likely caused by the low effective population size of the horse Y chromosome due to a strong variation in male reproductive success. This may be due to the polygynous breeding patterns in wild horses and to a stronger bottleneck in male horses during domestication and might be further exacerbated by the intensive breeding practices in this species [20], [21].

The samples used in our study derive from purebred modern, mainly European, breeds, which have undergone intensive selection for particular traits during the past two centuries [30]. The refinement was mainly achieved through the disproportionate use of selected popular stallions and their descendants that were crossed to local mares. With the use of pedigree information, available in studbooks and open access databases, we inferred the impact of the upgrading process on the male horse population. We find that only a limited number of founders contribute to the extant horse haplotypes (Fig. 3B, Table 1).

Based on the pedigree information, we traced the effects of three introgression waves (the Neapolitan, the Oriental and the English waves) on NRY markers. The importance of the Thoroughbred in the English wave is clearly seen through the spread of HT3. In the “Original Arabians”, the Neapolitan horse and the central and Eastern European founders, the proportion of HT2 is about 50%. In founders from northern Europe, i.e. Iceland, Norway and the British Isles, and the Iberian Peninsula the frequency of HT2 was very low. The distribution of HT2 is consistent with the movement of stallions from the Middle East to Central and Western Europe via the Neapolitan and Oriental waves (Fig. 3C, Fig. S10). The high proportion of HT2 in Central European horses may also be derived from earlier introgression of horses from the Middle East or even from ancient colonization [20].

Only three northern European horse breeds, Icelandic horses, Shetland ponies and Norwegian Fjord horses, were either not or were hardly subjected to these introgression waves and were therefore able to maintain autochthonous Y chromosome variants. These breeds have a comparatively isolated history. Due to the specific geographical and social structures in northern areas, locally adapted breeds were presumably of a higher value than imported animals from Central Europe and the Near East. In the case of Icelandic horses, the import of horses was restricted since 930 A.D. and has been prohibited since 1909 [30]. The three breeds are not necessarily closely related to one another. Icelandic and Norwegian Fjord horses branch from the root of modern breeds, when using 46244 autosomal loci [53]. As we only detected the ubiquitous HT1, little can be deduced about the ancestry of the Barb, the Iberian breeds, or the Swedish Coldblood Trotter.

The history of the domestic horse is marked by recurrent adjustment to changing socio-economic needs. Over the course of the last two centuries, when most modern breeds were established, the horse has been undergoing a transition from a working animal also used by the military use towards a domestic animal used in leisure and sports activities. This has largely been achieved through the use of a few, very popular, sires that have been extensively shifted among breeds. Due to the breeding practices during the last 200 years, “popular sires” and their sons have fathered an inordinate amount of offspring and replaced local Y chromosomes. Unique variants can only be found in a few northern European breeds. The restricted genetic diversity of the modern horse Y chromosome reflects what has survived through the species’ dynamic history.

### Conclusions

We describe the first polymorphic markers on the paternally inherited part of the Y chromosome of the domestic horse. We have used the new markers to investigate the paternal gene flow between horse populations and breeds. We document the influence of popular sires, particularly a single Thoroughbred stallion line, on many extant horse breeds. Our data on male lineages complement the information on the well established maternal mtDNA lineages and enhance the genetic documentation of the history and dynamics of modern horse breeds. The new polymorphic markers and haplotypes enable horse breeding practices to be monitored and verified. Although six haplotypes do not offer a high resolution, this study provides a first backbone phylogeny for deeper population genetic studies of the Y chromosome variation in horses.

### Data Deposition

BAC sequences have been deposited in Genbank (http://www.ncbi.nlm.nih.gov/genbank) under accession numbers JX565700–JX565709, sequence alignments under accession numbers JX646942– JX647045. Illumina short read sequences generated in this study are available at the Sequence Read Archive (SRA) under the Accesssion number ERP001668 (http://www.ebi.ac.uk/ena/data/view/ERP001668 ). Y-chromosomal SNPs have been submitted to the NCBI dbSNP database: ss#711581504, ss#711581506, ss#711581507.

## Supporting Information

Figure S1 **Workflow of the experimental setup performed in this study.**
(PDF)

Figure S2 **Male specificity of long range PCR products.**
(PDF)

Figure S3 **Information on the polymorphic site YXX_24I23 - Pos 25345.**
(PDF)

Figure S4 **Information on the polymorphic site YE3 - Pos 10594.**
(PDF)

Figure S5 **Information on the polymorphic site YE3 - Pos 1007–12040.**
(PDF)

Figure S6 **Information on the polymorphic site YE17.1 - Pos 1277.**
(PDF)

Figure S7 **Information on the polymorphic site YM23 - Pos 4161.**
(PDF)

Figure S8 **Information on the polymorphic site YE3 - Pos 11076–12042 deleted.**
(PDF)

Figure S9 **Sequence structures of the YE3 regions, giving information on gene conversion and deletion events at this region.**
(PDF)

Figure S10 **Details on the import of Original Arabian stallions.**
(PDF)

Table S1 **Nomenclature and Y-chromosomal localisation of the BAC clones selected for 454 sequencing.**
(DOCX)

Table S2 **BAC 454 Sequencing information.**
(DOCX)

Table S3 **Long range PCR information including locus identifiers, Primer sequences and amplicon sizes.**
(DOCX)

Table S4 **Sample information for the pooled Illumina Seq.**
(DOCX)

Table S5 **Primer sequences for Y-SNP verification.**
(DOCX)

Table S6 **Microsatellite analysis information.** Microsatellite PCR primers, labels and observed alleles in E. caballus (n = 100, 42 different breeds) and E. przewalskii (n = 3).
(DOCX)

Table S7 **Haplotype distribution for the breeds under investigation.** Data table for the phylogeography in Fig. 3A (breed information, number of samples and observed haplotpyes).
(DOCX)

Table S8 **Primer sequences for the Sequenom analysis and the screening of the deletions.**
(DOCX)

Table S9 **Alignments and Polymorphic sites.**
(DOCX)

Table S10 **Y-chromosomal dbSNPs.**
(DOCX)

Table S11 **Y-chromosomal haplotypes.**
(DOCX)
