# Supplementary material for: Identification of Genetic Variation on the Horse Y Chromosome and the Tracing of Male Founder Lineages in Modern Breeds
Source: PLoS One. 2013 Apr 3;8(4):e60015. doi: 10.1371/journal.pone.0060015 (PMC3616054; doi:10.1371/journal.pone.0060015)

Fig. S1. The experimental setup performed in this work is shown in a flow chart.

1. Identification of Y-chromosomal markers (red)
   - Generation of Y-chromosomal references
   - Illumina Seq of LRP products in 3 pools to identify Y-chromosomal variants
   - Validation of candidate polymorphisms by capillary sequencing of the candidate region in each horse from the seq-pools
   - Reconstruct the resultant haplotypes and visualize their phylogenetic relationship
2. Distribution of Y-chromosomal haplotypes (green)
   - Screening of Y-chromosomal haplotypes in various domestic horse breeds using the Sequenom MassARRAY iPLEX system. An additional haplotype (HT6) was discovered in this step, due to the impossibility of getting screening results for a particular locus in Shetland ponies (YE3 - Pos 1007-12040). Amplification of this region with walking primers led to the Identification of the 966 bp deletion (Y_E3.1.11076-12042del). A single individual carrying HT6 and a Przewalski horse harbouring an alternative Przewalski haplotype (as described in Wallner et al., 2004) were included in the SNP-validation approach („extended dataset").
   - Phylogeography
   - Microsatellite analysis was performed in a subset of 100 horses representig all domestic and Przewalskis horse haplotypes (including the individuals from the seqpools)
   - Trace the contribution of domestic horse founders in a subset of horses that have a well documented pedigree

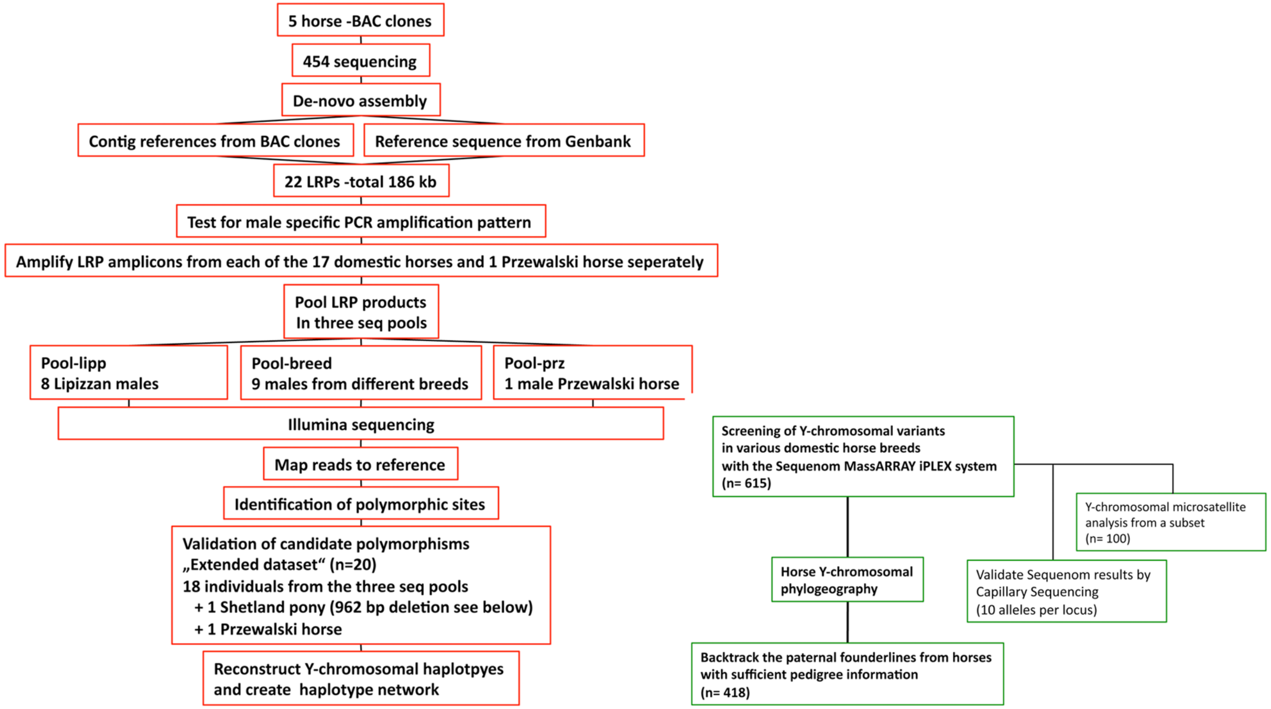

Supplement: Figure S1 — Workflow of the experimental setup performed in this study. (PDF) [file pone.0060015.s001.pdf]
