# Supplementary material for: Identification of Genetic Variation on the Horse Y Chromosome and the Tracing of Male Founder Lineages in Modern Breeds
Source: PLoS One. 2013 Apr 3;8(4):e60015. doi: 10.1371/journal.pone.0060015 (PMC3616054; doi:10.1371/journal.pone.0060015)

Fig. S2. Male specificity of long range PCR products.

LRP products amplified from male (m) and female (f) genomic DNA and no template control amplification (-) are seperated on a 0,7 % agarosegel. Gene Ruler DNAlatter mix (Thermo Scientific, #SM0331) was used as size standard. Amplicon identification is listed on the bottom of each image. Primers used are listed in Table S3. In case of multiple PCR products the Y chromosome specific amplicon (indicated by a red arrow) was isolated from the agarosegel prior NGS library preparation.

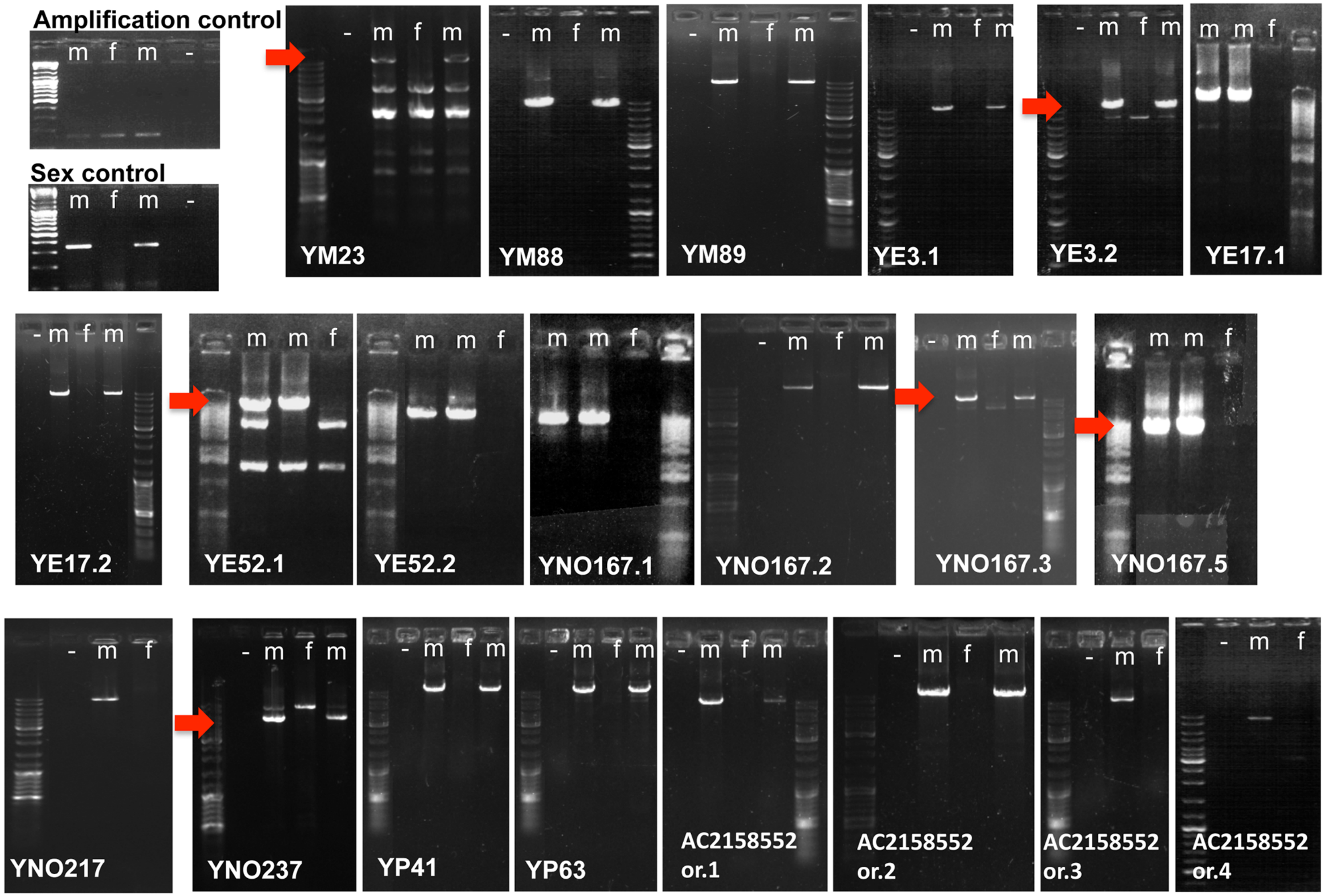

Supplement: Figure S2 — Male specificity of long range PCR products. (PDF) [file pone.0060015.s002.pdf]
