# Supplementary material for: Identification of Genetic Variation on the Horse Y Chromosome and the Tracing of Male Founder Lineages in Modern Breeds
Source: PLoS One. 2013 Apr 3;8(4):e60015. doi: 10.1371/journal.pone.0060015 (PMC3616054; doi:10.1371/journal.pone.0060015)

Fig. S3. Information on the polymorphic site YXX_24I23 - Pos 25345

(a) Sequence alignment (HT1,2,3,5,6,HTPrz1,2; HT4; Reference YXX_24I23 from genbank) with primer positions underlined. Positions identical to the sequence in the first row are represented with a hyphen. The mutation leading to HT4 is marked. b) PCR products amplified from male (m) and female (f) genomic DNA and a no-template-control (-). c) Capillary sequence traces of the confirmed mutations in HT1,2,3,5,6 and HT4, respectively.

A

```
YXX_24I23HT1/2/3/5/6/HTPrz     CTGTTAAGTA ATCACGGTTT AACTTTTTCT CAGTAAAAGC TTATGTTCCC TCCGGCCTTT 25260
YXX_24I23HT4                   ---------- ---------- ---------- ---------- ---------- ----------
ReferenceYXX_24I23             ---------- ---------- ---------- ---------- ---------- ----------

YXX_24I23HT1/2/3/5/6/HTPrz     ATGTCTTAGC ATTTTAAAAA CCTGTGGAAG GATAAAATTA GTACCAAGAT AAATGAGGAT 25320
YXX_24I23HT4                   ---------- ---------- ---------- ---------- ---------- ----------
ReferenceYXX_24I23             ---------- ---------- ---------- ---------- ---------- ----------

YXX_24I23HT1/2/3/5/6/HTPrz     AGATGGGGAA AAGGTTGAAA ATACGTTTAA CGTAGTTTAT GTCTTTCGTA AAACTTAAGA 25380
YXX_24I23HT4                   ---------- ---------- ----A----- ---------- ---------- ----------
ReferenceYXX_24I23             ---------- ---------- ---------- ---------- ---------- ----------

YXX_24I23HT1/2/3/5/6/HTPrz     CTATTAACTG TGAGATGTGT AGATTCCAGA CCATATTTTT TATTGTTTAC TTTTAGGTTA 25440
YXX_24I23HT4                   ---------- ---------- ---------- ---------- ---------- ----------
ReferenceYXX_24I23             ---------- ---------- ---------- ---------- ---------- ----------

YXX_24I23HT1/2/3/5/6/HTPrz     AAAAAAACAA GCCATGTCAT GTTTTTAAAG TGAACGTTGT ATACTGCAGC CCAAAAGAAT 25500
YXX_24I23HT4                   ---------- ---------- ---------- ---------- ---------- ----------
ReferenceYXX_24I23             ---------- ---------- ---------- ---------- ---------- ----------
```

B

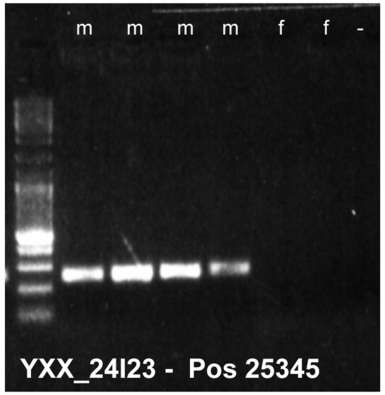

C

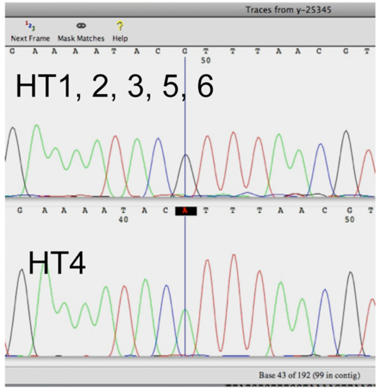

Supplement: Figure S3 — Information on the polymorphic site YXX_24I23 - Pos 25345. (PDF) [file pone.0060015.s003.pdf]
