# Supplementary material for: Identification of Genetic Variation on the Horse Y Chromosome and the Tracing of Male Founder Lineages in Modern Breeds
Source: PLoS One. 2013 Apr 3;8(4):e60015. doi: 10.1371/journal.pone.0060015 (PMC3616054; doi:10.1371/journal.pone.0060015)

Fig. S4. Information on the polymorphic site YE3 - Pos 10594

(a) Sequence alignment (HT1,2,4,5,6, HTPrz1,2; HT3; Reference from BAC clone E; homologous sequence on EcaX) with primer positions underlined. Positions identical to the sequence in the first raw are represented with a hyphen. The variant leading to HT3 is marked. b) PCR products amplified from male (m) and female (f) genomic DNA and a no-template-control (-) when placing the reverse primer in a Y-exclusive region (underlined.) (c) PCR products amplified from the same samples as in (B) with the reverse primer shifted downstream, leading to an amplification in females also. (d) Capillary sequence traces of the confirmed mutations in HT1,2,3,5,6 and HT3, respectively.

A

```
YE3_HT1/2/4/5/6/HTPrz  CCCTAATCCA TTTCAGTAAC TTCCCTCTGC TGAGCATCTA GGATTCCAGA AATGAAATCT 10504
YE3_HT3                ---------- ---------- ---------- ---------- ---------- ----------
YE3_BAC                ---------- ---------- ---------- ---------- ---------- ----------
ecaX:6050390- 6047628  ------c--- ---------- ---------- ---------- ----c----- ----------

YE3_HT1/2/4/5/6/HTPrz  GGGTCCAAGA TAAGTCTTTT GAAACATGTT GCCAAACTAC TCACCAGAAA AGCTGTCAGA 10564
YE3_HT3                ---------- ---------- ---------- ---------- ---------- ----------
YE3_BAC                ---------- ---------- ---------- ---------- ---------- ----------
ecaX:6050390- 6047628  ---------- ----c---c- ---------- ---------- ---------- -------t--

YE3_HT1/2/4/5/6/HTPrz  TTCGTAGTCT AATCAGCATT AGAACAAGTT AAGTGCTTAT TTCTCACAGT CTCACCCACA 10624
YE3_HT3                ---------- ---------- ---------. ---------- ---------- ----------
YE3_BAC                ---------- ---------- ---------- ---------- ---------- ----------
ecaX:6050390- 6047628  ---cc----- ---------- ---------. ---------- ---------- ----------

YE3_HT1/2/4/5/6/HTPrz  ATGGGCACTG CTCTTCTATT AGATCTCTGT CAACTTGACA GT.AAAACCT AGTGTCTCAC 10683
YE3_HT3                ---------- ---------- ---------- ---------- --.------- ----------
YE3_BAC                ---------- ---------- ---------- ---------- --.------- ----------
ecaX:6050390- 6047628  ---------- ---------- ------t--c ---------- --c------- ----------

YE3_HT1/2/4/5/6/HTPrz  TTTTAAA... .CTTGTAGGT CCTAGTTAAA CTCTTTTT.. ....ATATGC TTGTTTCCTA 10733
YE3_HT3                -------... .--------- ---------- --------.. ....------ ----------
YE3_BAC                -------... .--------- ---------- --------.. ....------ ----------
ecaX:6050390- 6047628  -------taa g----c---- ----c----- -aTC--g-Tt aaca------ ----------

YE3_HT1/2/4/5/6/HTPrz  TATGTACTTC TCA....... .......... CTGTCCCTTT CATCCAACTG CCTACTGGGG 10776
YE3_HT3                ---------- ---....... .......... ---------- ---------- ----------
YE3_BAC                ---------- ---....... .......... ---------- ---------- ----------
ecaX:6050390- 6047628  -t--c----- ---ttttagg aactgcctat t---g----- ---------t t---------

YE3_HT1/2/4/5/6/HTPrz  TATAAATTCC TTTCTTTACA TCAGCTACTG CTAAACCAGC TTTGTTACTA TACACCTGCT 10836
YE3_HT3                ---------- ---------- ---------- ---------- ---------- ----------
YE3_BAC                ---------- ---------- ---------- ---------- ---------- ----------
ecaX:6050390- 6047628  --c------- c---c----- --t-t----- ---------- ---------- --t-t-----
```

B

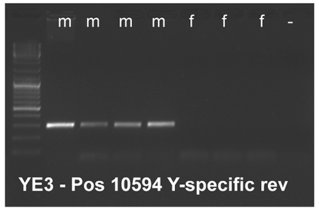

C

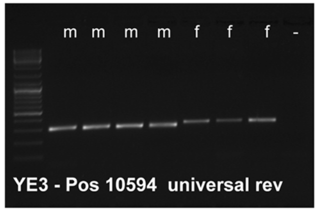

D

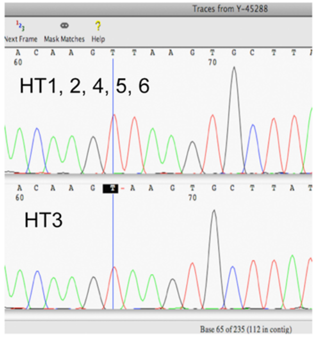

Supplement: Figure S4 — Information on the polymorphic site YE3 - Pos 10594. (PDF) [file pone.0060015.s004.pdf]
