# Supplementary material for: Identification of Genetic Variation on the Horse Y Chromosome and the Tracing of Male Founder Lineages in Modern Breeds
Source: PLoS One. 2013 Apr 3;8(4):e60015. doi: 10.1371/journal.pone.0060015 (PMC3616054; doi:10.1371/journal.pone.0060015)

Fig. S5. Information on the polymorphic site YE3 - Pos 1007-12040

(a) Sequence alignment (HT1,2,3,4 HTPrz1,2; HT5; HT6; Reference from BAC clone E; homologous sequence on EcaX) with primer positions underlined. Positions identical to the sequence in the first raw are represented with a hyphen. The variants explained as gene cenversion leading to HT5 are marked . b) PCR products amplified from male (m) and female (f) genomic DNA and a no-template-control (-) when placing the reverse primer in a Y-exclusive region (underlined.) (c) Capillary sequence traces of the polymorphic region HT1,2,3,4 and HT5, respectively.

A

```
YE3_HT1/2/3/4/HTPrz       TGGTGAACAT ATTCTTGCAT CCTG.TTCTG TCCCCCAACA CACGTCAACA GCTCTGTCTA GTTTGTTTGC TATTGCCTCC CTAGGGCTGG 10985
YE3_HT5                   ---------- ---------- ----.----- ---------- ---------- ---------- ---------- ---------- ----------
YE3_HT6                   ---------- ---------- ----.----- ---------- ---------- ---------- ---------- ---------- ----------
YE3-BAC                   ---------- ---------- ----.----- ---------- ---------- ---------- ---------- ---------- ----------
ecaX:6050390- 6047628     c--------- ---------c ----a----c -------g-- --t-a----g -------... .---a----- -gc------- -c--------

YE3_HT1/2/3/4/HTPrz       GTAGCTATTT TATGAATGAG TACTTCAGTG TCAGCATCCT CATGAGATCT TCCAGTGATG CCCATCCAGA TGCACTTCAA GTCAACTTGA 11075
YE3_HT5                   ---------- ---------- c--c------ ---------- ---------- ---------- ---------- ----g----- ----------
YE3_HT6                   ---------- ---------- ---------- ---------- ---------- ---------- ---------- ---------- ----------
YE3-BAC                   ---------- ---------- ---------- ---------- ---------- ---------- ---------- ---------- ----------
ecaX:6050390- 6047628     --g------- ---------- c--c------ ---------- ---------- ---------- ---------- ----g----- ----------

YE3_HT1/2/3/4/HTPrz       TTCAATAAAC CCAAACCCCT CATTGGCCCT TGTGCACCAG GCACTACACT AAGCAGGCTC TGGTAAGACA GAGTTCACAG ACAGCATTCA 11175
YE3_HT5                   ---------- ---------- -----t---- ---------- ---------- --------c- g---g---g- ------g-t- g---------
YE3_HT6                   .......... .......... .......... .......... .......... .......... .......... .......... ..........
YE3-BAC                   ---------- ---------- ---------- ---------- ---------- ---------- ---------- ---------- ----------
ecaX:6050390- 6047628     ---------- ---------- -----t---- ---------- ---------- --------c- g---g---g- ------g-t- g---------

YE3_HT1/2/3/4/HTPrz       CTAAGCTGGT GCTTACCCCA CAGAAGAGAT GGTCAGTAAG CCAATGAGC. AGCCCAGTGT TT.GGGGGCT CTGGCAG... ........AG 11242
YE3_HT5                   ---------- ---------- ----C-G-.. ...-G----- ----C----C ---------- --T------- -------CCC TACCACAG--
YE3_HT6                   .......... .......... .......... .......... .......... .......... .......... .......... ..........
YE3-BAC                   ---------- ---------- ---------- ---------- ---------. ---------- --.------- -------... ........--
ecaX:6050390- 6047628     ---------- ---------- ----c-g-.. ...-g----- ----c----C ---------- --T------- -------ccc taccacag-

YE3_HT1/2/3/4/HTPrz       ACAATACGGG AGATAGAAGA AGAGAAGAAC CCCAGTCAGC CTGGGGGCTG GGTGATGATA CCCAGAGATG GTCTATAACT CCTGTCTTAC 11332
YE3_HT5                   ------T-A- ---C-C---- ---------- --T-A----- ---------- ---------T ---------- ---------- ----------
YE3_HT6                   .......... .......... .......... .......... .......... .......... .......... .......... ..........
YE3-BAC                   ---------- ---------- ---------- ---------- ---------- ---------- ---------- ---------- ----------
ecaX:6050390- 6047628     ------t-a- ---c-c---- ---------- --t-a----- ---------- ---------t ---------- ---------- ----------

YE3_HT1/2/3/4/HTPrz       CGCTGCAGAT CAAGCTTCCC TTACCATCA. .....GTGGC CTAAGCCTAA CTGAATTCTG TGAGAACACA CATCCTGGGT GCCCTAGGAG 11416
YE3_HT5                   ---------- ---------- ---------. .....----- ---------- ---------- ---------- ---------- ----------
YE3_HT6                   .......... .......... .......... .......... .......... .......... .......... .......... ..........
YE3-BAC                   ---------- ---------- ---------. .....----- ---------- ---------- ---------- ---------- ----------
ecaX:6050390- 6047628     -c----g--- ---------- ---------c CATCA----- -----t---- ------c-c- ---------- ---------- ----------
```

B

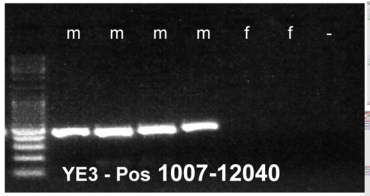

C

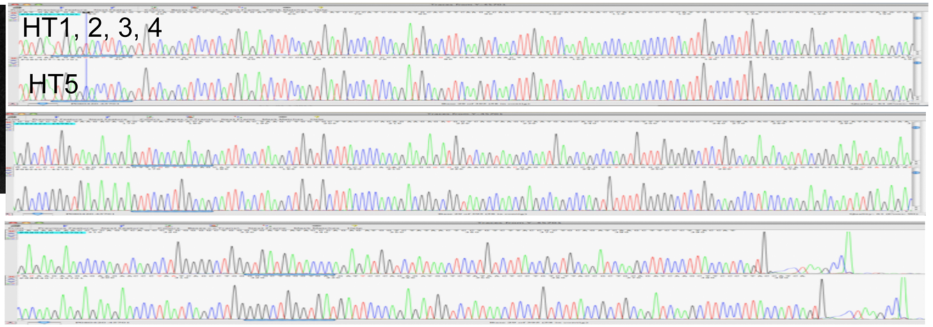

Supplement: Figure S5 — Information on the polymorphic site YE3 - Pos 1007–12040. (PDF) [file pone.0060015.s005.pdf]
