# Supplementary material for: Identification of Genetic Variation on the Horse Y Chromosome and the Tracing of Male Founder Lineages in Modern Breeds
Source: PLoS One. 2013 Apr 3;8(4):e60015. doi: 10.1371/journal.pone.0060015 (PMC3616054; doi:10.1371/journal.pone.0060015)

Fig. S6. Information on the polymorphic site YE17.1 - Pos 1277

(a) Sequence alignment (HT1,4,5,6, HTPrz1,2; HT2,3; Reference from BAC clone E; homologous sequence on EcaX) with primer positions underlined. Positions identical to the sequence in the first raw are represented with a hyphen. The mutation leading to HT2/3 is marked. (b) PCR products amplified from male (m) and female (f) genomic DNA and a no-template-control (-). (c) Capillary sequence traces of the confirmed mutations in HT1,4,5,6 and HT2,3 respectively.

A

```
YE17_HT1/4/5/6/HTPrz   TCTGAGGTGG TGAGAGAATC ATACTTGAGA GCACATGTTG CAAAGCCAAG ATGCAGGGCC 1166
YE17_HT2/3             ---------- ---------- ---------- ---------- ---------- ----------
YE17-BACcontig         ---------- ---------- ---------- ---------- ---------- ----------
ecaX:6089749-6071249   --c------- -cca------ ---------- -agtg----- ---------- ----------

YE17_HT1/4/5/6/HTPrz   TAAGTTGTTC GCAGAGAGCA AGATTACCTC CTGGGACAAC TAGAGCAGGA CCCAGAGGTG 1126
YE17_HT2/3             ---------- ---------- ---------- ---------- ---------- ----------
YE17-BACcontig         ---------- ---------- ---------- ---------- ---------- --c-------
ecaX:6089749-6071249   ---------t --------g- ---------- ---------- -c-g------ ----------

YE17_HT1/4/5/6/HTPrz   CTGCCTTTGC AACAGAGCTA GGCTTCCATG AA....TGAC TCTCGAGTTC TACATTCACA 1282
YE17_HT2/3             ---------- ---------- ---------- --....---- ---------- ----A-----
YE17-BACcontig         ---------- ---------- ---------- --....---- ---------- ----------
ecaX:6089749-6071249   -------c-- --------c- ---------- --cagt---- ---------- c---------

YE17_HT1/4/5/6/HTPrz   GTTTGAGATT ACTGTTTAGA AAGCACACTT CTGTTGGTTG CAGGCGTGAA TAACAGTGAT 1342
YE17_HT2/3             ---------- ---------- ---------- ---------- ---------- ----------
YE17-BACcontig         ---------- ---------- ---------- ---------- ---------- ----------
ecaX:6089749-6071249   -------g-- -gga------ -----t---- -c-------t cgggtgtgag tcac--....

YE17_HT1/4/5/6/HTPrz   TGTATAAAGT GTAGAATCAC TTATTAGAAA TCAGAGACAA TCTATCTGTT GTTCAGCCGG 1402
YE17_HT2/3             ---------- ---------- ---------- ---------- ---------- ----------
YE17-BACcontig         ---------- ---------- ---------- ---------- ---------- ----------
ecaX:6089749-6071249   .......... .......... .......... ......---g --------cc c-------t-

YE17_HT1/4/5/6/HTPrz   GTGGA...CA CTGGACACCA CCAGTCAGG. CTGTCCGATG CTGGAGAACA ACGATGCTAC 1458
YE17_HT2/3             -----...-- ---------- ---------. ---------- ---------- ----------
YE17-BACcontig         -----...-- ---------- ---------. ---------- ---------- ----------
ecaX:6089749-6071249   ----gatg-- -----t---- --g------g t----t---- ---------- -a--------
```

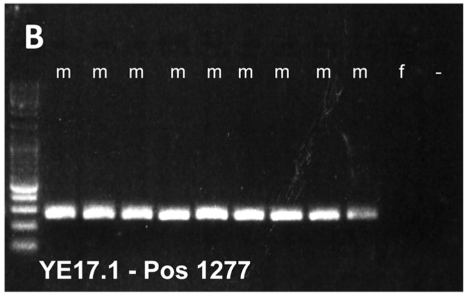

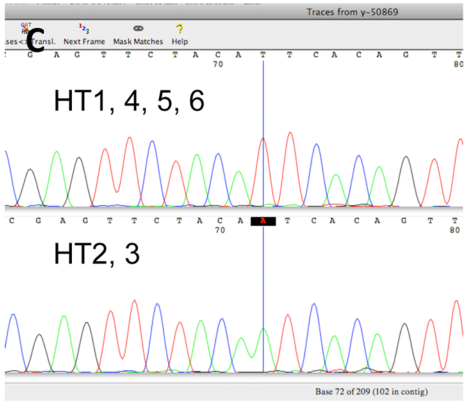

Supplement: Figure S6 — Information on the polymorphic site YE17.1 - Pos 1277. (PDF) [file pone.0060015.s006.pdf]
