# Supplementary material for: Identification of Genetic Variation on the Horse Y Chromosome and the Tracing of Male Founder Lineages in Modern Breeds
Source: PLoS One. 2013 Apr 3;8(4):e60015. doi: 10.1371/journal.pone.0060015 (PMC3616054; doi:10.1371/journal.pone.0060015)

Fig. S7. Information on the polymorphic site YM23 - Pos 4161

(a) Sequence alignment (HT1,2,3,4,5,6; HTPrz1; HTPrz2; Reference from BAC clone E) with primer positions underlined. Positions identical to the sequence in the first raw are represented with a hyphen. The mutation leading to HTPrz2 is marked. (b) PCR products amplified from male (m) and female (f) genomic DNA and a no-template-control (-). (c) Capillary sequence traces of the confirmed mutations in HT1,2,3,4,5,6 and HTPrz1 and HTPrz2 respectively.

A

```
YM23_HT1/2/3/4/5/6     TTTCGGATTG ATGATCTCAT GGAGCCTTCA CCATCTTGTC CACTTCGGCA GAGAGACTAC 3840
YM23_HT1Prz2           ---------- ---------- ---------- ---------- ---------- ----------
YM23_HT1Prz1           ---------- ---------- ---------- ---------- ---------- ----------
YM23-BACcontig         ---------- ---------- ---------- ---------- ---------- ----------

YM23_HT1/2/3/4/5/6     AGACCCCTTT ACTGAGGAGA AAAGTGAGAC ACCTTTCATA GCGATATCCT AATCTGCAAT 3900
YM23_HT1Prz2           ---------- ---------- ---------- ---------- ---------- ----------
YM23_HT1Prz1           ---------- ---------- ---------- ---------- ---------- ----------
YM23-BACcontig         ---------- ---------- ---------- ---------- ---------- ----------

YM23_HT1/2/3/4/5/6     CTGCGTGCCT TTAAACGGCA ACACCTCCCA CGCAGTCTTA CCCTTCCACC TAGCTTCAGG 3960
YM23_HT1Prz2           ---------- ---------- ---------- ---------- ---------- ----------
YM23_HT1Prz1           ---------- ---------- ---------- ---------- ---------- ----------
YM23-BACcontig         ---------- ---------- ---------- ---------- ---------- ----------

YM23_HT1/2/3/4/5/6     AACTCAGTCT TCCCTGCTAG CCCTTGGGGT TCTGAGCTTG CTCCCTCAGA AGAGATAGGA 4020
YM23_HT1Prz2           ---------- ---------- ---------- ---------- ---------- ----------
YM23_HT1Prz1           ---------- ---------- ---------- ---------- ---------- ----------
YM23-BACcontig         ---------- ---------- ---------- ---------- ---------- ----------

YM23_HT1/2/3/4/5/6     CCTTGCCTTT AGGTTTCCCG CTCCCGCCTG CTAGACCCGC CGGTGCCCAG CCCCCGGAGT 4080
YM23_HT1Prz2           ---------- ---------- ---------- ---------- ---------- ----------
YM23_HT1Prz1           ---------- ---------- ---------- ---------- ---------- ----------
YM23-BACcontig         ---------- ---------- ---------- ---------- ---------- ----------

YM23_HT1/2/3/4/5/6     CCTGGTGAAT GAGCTACAGA GGCTCCAGGG AATTGAACTG CGGCGAAAGC AGGTACACGT 4140
YM23_HT1Prz2           -----C---- ---------- ---------- ---------- ---------- ----------
YM23_HT1Prz1           -----C---- ---------- ---------- ---------- ---------- ----------
YM23-BACcontig         ---------- ---------- ---------- ---------- ---------- ----------

YM23_HT1/2/3/4/5/6     CCCACGTGGA ACACAGATTT GCAGACCTTC TGCGACGAAA GCTATGAGGC ACACGCCACA 4200
YM23_HT1Prz2           ---------- ---------- a--------- ---------- ---------- ----------
YM23_HT1Prz1           ---------- ---------- g--------- ---------- ---------- ----------
YM23-BACcontig         ---------- ---------- ---------- ---------- ---------- ----------
```

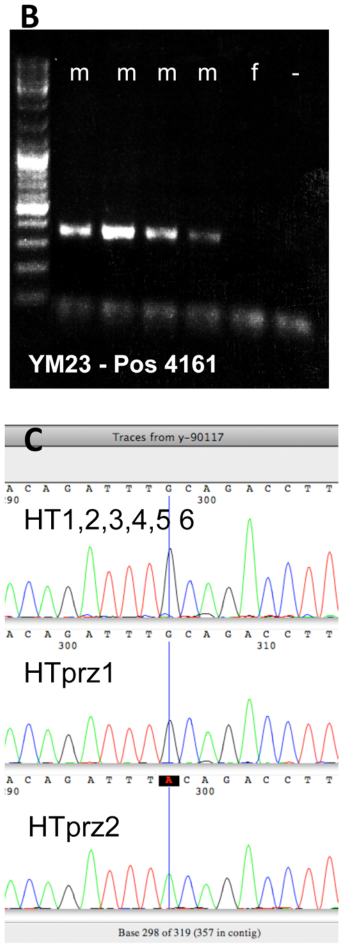

Supplement: Figure S7 — Information on the polymorphic site YM23 - Pos 4161. (PDF) [file pone.0060015.s007.pdf]
