# Supplementary material for: Identification of Genetic Variation on the Horse Y Chromosome and the Tracing of Male Founder Lineages in Modern Breeds
Source: PLoS One. 2013 Apr 3;8(4):e60015. doi: 10.1371/journal.pone.0060015 (PMC3616054; doi:10.1371/journal.pone.0060015)

Fig. S8. Information on the polymorphic site YE3 - Pos 11076-12042 deleted

(a) Sequence region and primer position leading to a 2010 bp amplicon in HT1,2,3,4,5 and a 1044 bp product in HT6 (Shetland ponies). (b) Alignment (X-, Y-homologue) of the Y-specific reverse primer position (arrow). c) PCR products amplified from male HT1,2,6 (m) and female (f) genomic DNA and a no-template-control (-) when using primers from Table S10.

A

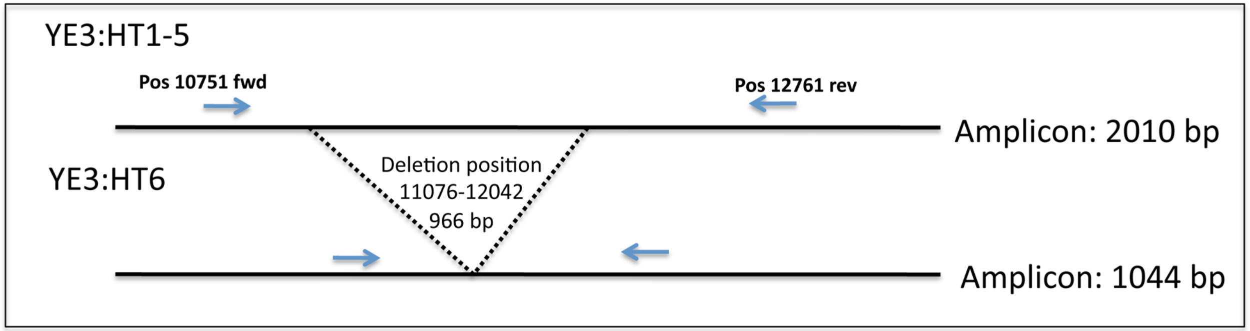

B

```
YE3_HT1/2/3/4/5/6         CTGTCCCTTTCATCCAACTGCCTACTGGGG
ecaX:6050390- 6047628     TTG-G--------------TT---------
```

C

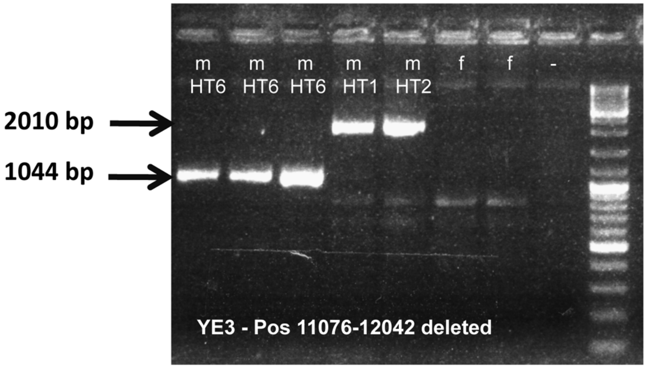

Supplement: Figure S8 — Information on the polymorphic site YE3 - Pos 11076–12042 deleted. (PDF) [file pone.0060015.s008.pdf]
