# Supplementary material for: Identification of Genetic Variation on the Horse Y Chromosome and the Tracing of Male Founder Lineages in Modern Breeds
Source: PLoS One. 2013 Apr 3;8(4):e60015. doi: 10.1371/journal.pone.0060015 (PMC3616054; doi:10.1371/journal.pone.0060015)

Fig. S9. Sequence structures of the YE3 regions, giving information on gene conversion and deletion events at this region.

Upper panel: Sequences homologous to the Y chromosome are drawn in orange (Y) and green (X). Numbers on the Y correspond to the position in the alignment whencomparing with HT1 (for Y) or to the chromosome position on the horse X (Broad/equcab2; http://genome.ucsc.edu/). Blue stripes indicate a dinucleotide microsatellite.

Lower panel: Structural changes on this locus, leading to HT5 (gene conversion), HT3 (putative gene conversion) and HT6 (deletion).

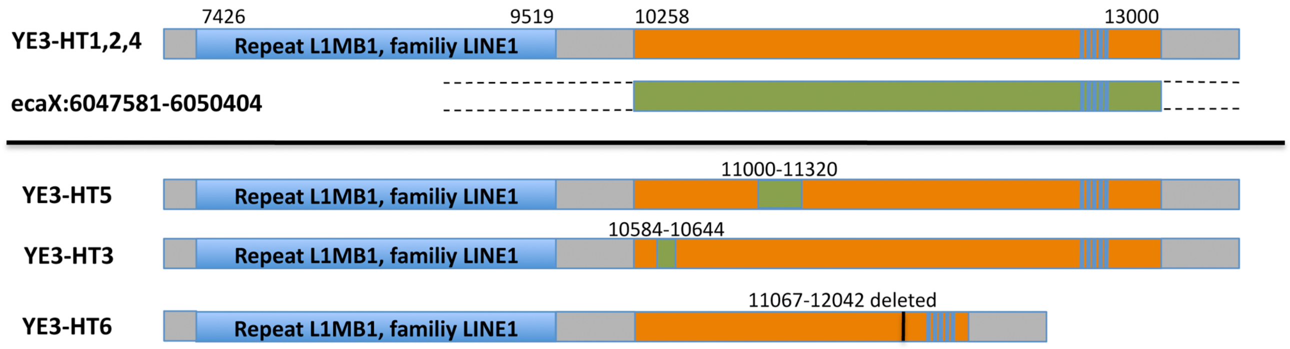

Supplement: Figure S9 — Sequence structures of the YE3 regions, giving information on gene conversion and deletion events at this region. (PDF) [file pone.0060015.s009.pdf]
