# Supplementary material for: Identification of Genetic Variation on the Horse Y Chromosome and the Tracing of Male Founder Lineages in Modern Breeds
Source: PLoS One. 2013 Apr 3;8(4):e60015. doi: 10.1371/journal.pone.0060015 (PMC3616054; doi:10.1371/journal.pone.0060015)

Fig. S10. Details on the import of Original Arabian stallions

Documented imports (with date) of Arabian horses carrying either HT1 (blue) or HT2 (yellow) to Central European studs (Oriental wave) and the spreading of the English thoroughbred (HT3 – red).

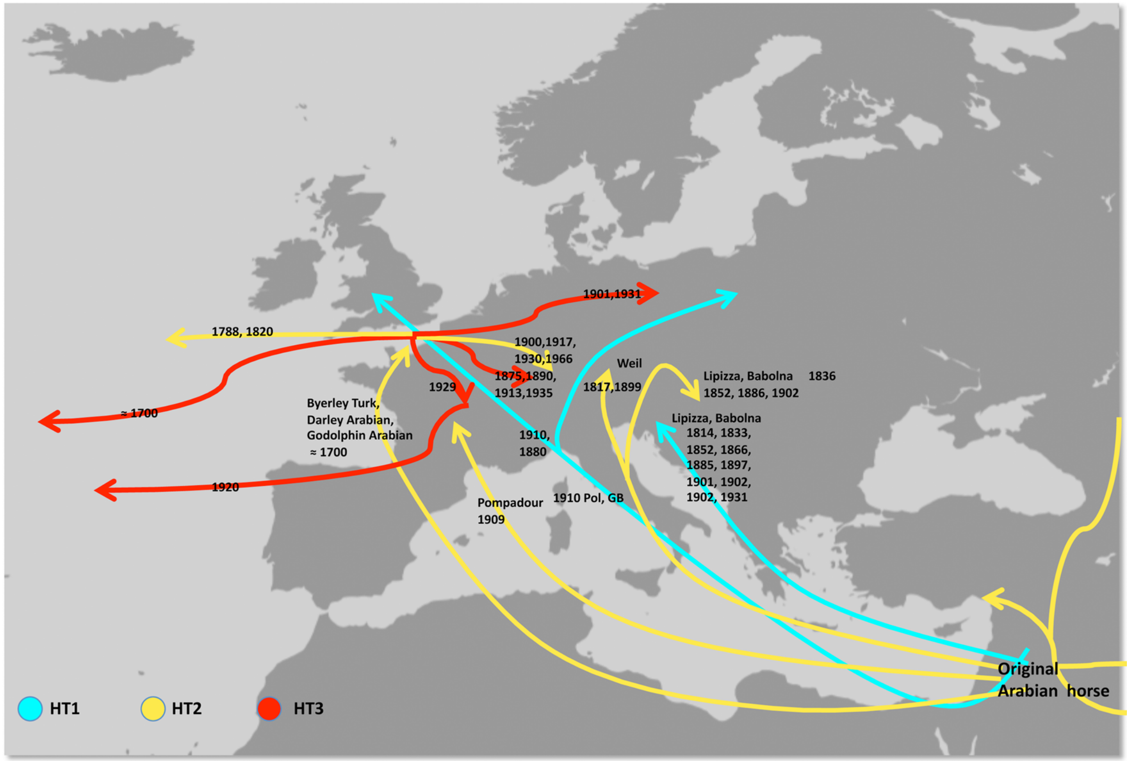

Supplement: Figure S10 — Details on the import of Original Arabian stallions. (PDF) [file pone.0060015.s010.pdf]
