# Supplementary material for: Identification of Genetic Variation on the Horse Y Chromosome and the Tracing of Male Founder Lineages in Modern Breeds
Source: PLoS One. 2013 Apr 3;8(4):e60015. doi: 10.1371/journal.pone.0060015 (PMC3616054; doi:10.1371/journal.pone.0060015)
Supplement: Table S1 — Nomenclature and Y-chromosomal localisation of the BAC clones selected for 454 sequencing. (DOCX) [file pone.0060015.s011.docx]

### Table S1. Nomenclature and Y-chromosomal localisation of the BAC clones selected for 454 sequencing

| **BAC/ selection marker after Raudsepp et al., 2004 [17]** | **short name** | **Bac position on ecaY after Raudsepp et al.,2004** |
| --- | --- | --- |
| 1056F3/Y2B17 | E | Contig IV |
| 180F9/Y3B12, Y3B19 | N | Contig III |
| 927H5/Y3B12, Y3B19 | O | Contig III |
| 601D9/ Y3B8 | M | Contig I |
| 616B11/Sry | P | Contig I |
