# Supplementary material for: Identification of Genetic Variation on the Horse Y Chromosome and the Tracing of Male Founder Lineages in Modern Breeds
Source: PLoS One. 2013 Apr 3;8(4):e60015. doi: 10.1371/journal.pone.0060015 (PMC3616054; doi:10.1371/journal.pone.0060015)
Supplement: Table S2 — BAC 454 Sequencing information. (DOCX) [file pone.0060015.s012.docx]

### Table S2. BAC 454 Sequencing information

454 Sequencing of 5 BAC clones yielded 155 510 reads with a mean length of 237 bp after trimming. The number of de-novo assembled contigs ranged from 25 (clone P) to 117 per clone (clone N-O) with an average length of 2700 bp. Taking only contigs >5 kb on consideration, 22 contigs were selected for further analysis. 11 contigs were not suitable for LRP and SNP screen due to a high proportion of repetitive elements.

| **path** | **BAC-N/O trimmed** |  |  | **BAC-E trimmed** |  |  | **BAC-M trimmed** |  |  | **BAC-P trimmed** |  |  |
| --- | --- | --- | --- | --- | --- | --- | --- | --- | --- | --- | --- | --- |
| **runMetrics** |  |  |  |  |  |  |  |  |  |  |  |  |
| totalNumberOfReads | 70937 |  |  | 14037 |  |  | 41031 |  |  | 29505 |  | 155510 |
| totalNumberOfBases | 16669639 |  |  | 3310176 |  |  | 9912692 |  |  | 7171576 |  |  |
| numberSearches | 31182 |  |  | 8967 |  |  | 19208 |  |  | 14381 |  |  |
| seedHitsFound | 27632407 | 886.17 |  | 1860513 | 207.48 |  | 16930514 | 881.43 |  | 7625033 | 530.22 |  |
| overlapsFound | 2722650 | 87.31 | 9.85% | 165879 | 18.5 | 8.92% | 1633535 | 85.04 | 9.65% | 695628 | 48.37 | 9.12% |
| overlapsReported | 2624109 | 84.15 | 96.38% | 156794 | 17.49 | 94.52% | 1576964 | 82.1 | 96.54% | 669188 | 46.53 | 96.20% |
| overlapsUsed | 70171 | 2.25 | 2.67% | 10029 | 1.12 | 6.40% | 40658 | 2.12 | 2.58% | 24483 | 1.7 | 3.66% |
| estimatedGenomeSize | 7.1 MB |  |  | 1.2 MB |  |  | 4.1 MB |  |  | 3.0 MB |  |  |
| **consensusResults** |  |  |  |  |  |  |  |  |  |  |  |  |
| numAlignedReads | 60698 | 85.57% |  | 9527 | 67.87% |  | 34079 | 83.06% |  | 24812 | 84.09% |  |
| numAlignedBases | 13833745 | 82.99% |  | 2128091 | 64.29% |  | 7897434 | 79.67% |  | 5855676 | 81.65% |  |
| inferredReadError | 1.48% | 204303 |  | 1.46% | 31051 |  | 1.54% | 121867 |  | 1.50% | 88105 |  |
| numberAssembled | 33147 |  |  | 4999 |  |  | 18330 |  |  | 13881 |  |  |
| numberPartial | 27551 |  |  | 4528 |  |  | 15749 |  |  | 10931 |  |  |
| numberSingleton | 9049 |  |  | 4336 |  |  | 6423 |  |  | 4530 |  |  |
| numberRepeat | 616 |  |  | 25 |  |  | 184 |  |  | 30 |  |  |
| numberOutlier | 574 |  |  | 149 |  |  | 345 |  |  | 133 |  |  |
| numberTooShort | 0 |  |  | 0 |  |  | 0 |  |  | 0 |  |  |
| **largeContigMetrics** |  |  |  |  |  |  |  |  |  |  |  |  |
| numberOfContigs | 117 |  |  | 36 |  |  | 48 |  |  | 25 |  |  |
| numberOfBases | 223910 |  |  | 133291 |  |  | 119959 |  |  | 134571 |  |  |
| avgContigSize | 1913 |  |  | 3702 |  |  | 2499 |  |  | 5382 | 2699.2 |  |
| N50ContigSize | 6000 |  |  | 16432 |  |  | 10889 |  |  | 24938 |  |  |
| largestContigSize | 49282 |  |  | 36348 |  |  | 17571 |  |  | 29022 |  |  |
| Q40PlusBases | 220598 | 98.52% |  | 130945 | 98.24% |  | 118854 | 99.08% |  | 133552 | 99.24% |  |
| Q39MinusBases | 3312 | 1.48% |  | 2346 | 1.76% |  | 1105 | 0.92% |  | 1019 | 0.76% |  |
| **allContigMetrics** |  |  |  |  |  |  |  |  |  |  |  |  |
| numberOfContigs | 208 |  |  | 61 |  |  | 109 |  |  | 59 |  |  |
| numberOfBases | 259986 |  |  | 144290 |  |  | 142242 |  |  | 147427 |  |  |
