# Supplementary material for: Identification of Genetic Variation on the Horse Y Chromosome and the Tracing of Male Founder Lineages in Modern Breeds
Source: PLoS One. 2013 Apr 3;8(4):e60015. doi: 10.1371/journal.pone.0060015 (PMC3616054; doi:10.1371/journal.pone.0060015)
Supplement: Table S3 — Long range PCR information including locus identifiers, Primer sequences and amplicon sizes. (DOCX) [file pone.0060015.s013.docx]

### Table S3. Long range PCR information including locus identifiers, Primer sequences and amplicon sizes

| source | contig | contig length | passed, Accession number | Primersequences for long range PCR | Amplicon length | contig identifier | total length |
| --- | --- | --- | --- | --- | --- | --- | --- |
| BAC-M | M23 | 11490 bp | Yes, AC.No JX565700 | ecYM23-fwd: AGGAGTGGCCTGTCCCACTAG | 10880 bp | YM23 | 10880 bp |
|  |  |  |  | ecYM23-rev: GAAGCAATCCAAGTGCCCATC |  |  |  |
| BAC-M | M71 | 10889 bp | No - total interspersed repeats (47%) |  |  |  |  |
| BAC-M | M88 | 11602 bp | Yes, AC.No JX565701 | ecYM88-fwd: GGCACCTTGATGCCCTAGAC | 8508 bp | YM88 | 8508 bp |
|  |  |  |  | ecYM88-rev: ACTGCCTCCACTTCCTCTGC |  |  |  |
| BAC-M | M89 | 11393 bp | Yes, AC.No JX565702 | ecYM89-fwd: GTGATTCCAGGCCAGCCTAC | 9393 bp | YM89 | 9393 bp |
|  |  |  |  | ecYM89-rev: ATGGATGGCACTGATGGTTG |  |  |  |
| BAC-M | M95 | 17231 bp | No - total interspersed repeats (51%) |  |  |  |  |
| BAC-E | E3 | 16412 bp | Yes, AC.No JX565703 | ecYE3.1-fwd: GAGAGGCATGCAAACAGTGC | 9285 bp | YE3 | 14898 bp |
|  |  |  |  | ecYE3.1-rev: TCAGGGTCCATCCATGTTATTG |  |  |  |
|  |  |  |  | ecYE3.2-fwd: ACATTGGCTCCCATCAATCC | 8870 bp |  |  |
|  |  |  |  | ecYE3.2-rev: CTGCCCTCTGTGGTCATCTG |  |  |  |
| BAC-E | E5 | 11543 bp | No - total interspersed repeats (55 %) |  |  |  |  |
| BAC-E | E17 | 19520 bp | Yes, AC.No JX565704 | ecYE17.1-fwd: GCACCTCAGTGGAGGgTAGG | 11482 bp | YE17 | 18678 bp |
|  |  |  |  | ecYE17.1b-rev: TCTAGCTTGAGCCTTGCATGTG |  |  |  |
|  |  |  |  | ecYE17.2-fwd: CCTCCACACCGGCTTAGAACC | 9440 bp |  |  |
|  |  |  |  | ecYE17.2-rev: AAGACCAGGGAGACAGGTCCAC |  |  |  |
| BAC-E | E38 | 9790 bp | No - total interspersed repeats (63 %) |  |  |  |  |
| BAC-E | E52 | 19573bp | Yes, AC.No JX565705 | ecYE 52.1-fwd: CTCTGCCCACCCTACCTGAC | 11129 bp | YE52 | 17810 bp |
|  |  |  |  | ecYE 52.1b-rev: TGGCACGCTCCACTTCTGTG |  |  |  |
|  |  |  |  | ecYE 52.2b-fwd: CCATTCCACTGCTGCCTGAG | 7270 bp |  |  |
|  |  |  |  | ecYE 52.2-rev: GAGATGGCACACTGCCTCAGC |  |  |  |
| BAC-N/O | NO142 | 6716 bp | No - total interspersed repeats (67 %) |  |  |  |  |
| BAC-N/O | NO148 | 13640 bp | No - total interspersed repeats (57 %) |  |  |  |  |
| BAC-N/O | NO157 | 7863 bp | No - total interspersed repeats (41%) |  |  |  |  |
| BAC-N/O | NO167 | 49282 bp | Yes, AC.No JX565706 | ecYNO167.1b-fwd: GCACCACCTACGTGCATCAT | 9491 bp | YNO167.1to3 | 27711 bp |
|  |  |  |  | ecYNO167.1-rev: CAGGGGACATGACTGCACTG |  |  |  |
|  |  |  |  | ecYNO167.2-fwd: TGGTGAAGGGCCTGTCAAGT | 9590 bp |  |  |
|  |  |  |  | ecYNO167.2-rev: ATCCCCATGCACACAGACCT |  |  |  |
|  |  |  |  | ecYNO167.3-fwd: CACTCTGAGGGCAAGGATGG | 11849 bp |  |  |
|  |  |  |  | ecYNO167.3-rev: TGCCAGATAAGCGAGCTTCC |  |  |  |
|  |  |  |  | ecYNO167.5-fwd: CATTGCCATCAGCAAACCAAC | 8829 bp | YNO167.5 | 8830 bp |
|  |  |  |  | ecYNO167.5b-rev: TAGGCATTTGCTGGACATGG |  |  |  |
| BAC-N/O | NO213 | 6777 bp | No - total interspersed repeats (58%) |  |  |  |  |
| BAC-N/O | NO217 | 8727 bp | Yes, AC.No JX565707 | ecYNO217-fwd: GAAAGCATGCACTGGCCATAT | 7619 bp | YNO217 | 7619 bp |
|  |  |  |  | ecYNO217-rev: CCCGGAAAAGACTTCAACTGC |  |  |  |
| BAC-N/O | NO237 | 6855 bp | Yes, AC.No JX565708 | ecYNO237-fwd: TGAGCCACAGGATGGAAGGT | 5528 bp | YNO237 | 5528 bp |
|  |  |  |  | ecYNO237-rev: TCCGCTGGATAAATGCCAAG |  |  |  |
| BAC-P | P23 | 28935 bp | No - total interspersed repeats (84%) |  |  |  |  |
| BAC-P | P41 | 13378 bp | Yes, AC.No JX565709 | ecYP41-fwd: CTGTATGGTGGGGCAAACCTC | 10487 bp | YP41 | 10487 bp |
|  |  |  |  | ecYP41-rev: CATCCAACACTTGGCATTCCTG |  |  |  |
| BAC-P | P43 | 29022 bp | No - total interspersed repeats (84%) |  |  |  |  |
| BAC-P | P44 | 24938 bp | No - total interspersed repeats (83%) |  |  |  |  |
| BAC-P/ AC215855.2 | P63/ AC215855.2 | 14296 bp | Yes, AC. No AC215855.2 | ecYP63-fwd: CTGCCCGCACAGTGTAGAAG | 11086 bp | YP63 | 11086 bp |
|  |  |  |  | ecYP63-rev: TTCACCAGGGACCATCAGTG |  |  |  |
| AC215855.2 | AC215855.2 | 34930 bp | Yes | AC215855or.1-fwd: GATTTGGACCCACCACAGGAG | 10227 bp | YXX_24I23 | 34694 bp |
|  |  |  |  | AC215855or.1-rev: GCCACTCCAAGGTGTTGAGG |  |  |  |
|  |  |  |  | AC215855or.2-fwd: AGCGTCTTCAGCGACCTCAC | 10189 bp |  |  |
|  |  |  |  | AC215855or.2-rev: AAGGGCATCCTTCGTGTTGAC |  |  |  |
|  |  |  |  | AC215855or.3-fwd: ACGGAGGTTCCTTTCGAAGC | 10302 bp |  |  |
|  |  |  |  | AC215855or.3-rev: TACAGCCTGGATGCTGCTGA |  |  |  |
|  |  |  |  | AC215855or.4-fwd: TTAGGCGGTCCAGGGCTAAC | 8499 bp |  |  |
|  |  |  |  | AC215855or.4-rev: TGCCTTTTCAATGCCATTCC |  |  |  |
|  |  |  |  |  |  |  | total 186123 bp |
|  |  |  |  |  |  | after removing primer sequence | 185589 bp |
