# Supplementary material for: Identification of Genetic Variation on the Horse Y Chromosome and the Tracing of Male Founder Lineages in Modern Breeds
Source: PLoS One. 2013 Apr 3;8(4):e60015. doi: 10.1371/journal.pone.0060015 (PMC3616054; doi:10.1371/journal.pone.0060015)
Supplement: Table S4 — Sample information for the pooled Illumina Seq. (DOCX) [file pone.0060015.s014.docx]

### Table S4. Sample information for the pooled Illumina Seq

| **Sample number** | **species** | **breed** | **paternal line** | **Sample -source** | **ILLUMINA SEQU-pool** | |
| --- | --- | --- | --- | --- | --- | --- |
| BW-B1-B 97001 | E. caballus | Lipizzan | Incitato | EU Copernicusproject | 9353 | lipp |
| BW-D38-D 97038 | E. caballus | Lipizzan | Pluto | EU Copernicusproject | 9353 | lipp |
| BW-D39-D 97039 | E. caballus | Lipizzan | Maestoso | EU Copernicusproject | 9353 | lipp |
| BW-F4-T 97004 | E. caballus | Lipizzan | Conversano | EU Copernicusproject | 9353 | lipp |
| BW-F5-T 97005 | E. caballus | Lipizzan | Tulipan | EU Copernicusproject | 9353 | lipp |
| BW-L41-L 97041 | E. caballus | Lipizzan | Siglavy | EU Copernicusproject | 9353 | lipp |
| BW-T5-T 97005 | E. caballus | Lipizzan | Neapolitano | EU Copernicusproject | 9353 | lipp |
| BW-W51-W 98051 | E. caballus | Lipizzan | Favory | EU Copernicusproject | 9353 | lipp |
| BW-135 | E. caballus | Icelandic horse |  | Wallner | 9354 | breed |
| BW-19 | E. caballus | Thouroghbred |  | Dworak BGL | 9354 | breed |
| BW-23 | E. caballus | Warmblood (Trakehner) |  | Dworak BGL | 9354 | breed |
| BW-25 | E. caballus | Quarter horse |  | Dworak BGL | 9354 | breed |
| BW-51 | E. caballus | Shetland pony |  | Dworak BGL | 9354 | breed |
| BW-57 | E. caballus | Shire horse |  | Dworak BGL | 9354 | breed |
| BW-64 | E. caballus | Shagya Araber |  | Dworak BGL | 9354 | breed |
| BW-65 | E. caballus | Norwegian Fjord horse |  | Dworak BGL | 9354 | breed |
| BW-XX | E. caballus | Arabian XX |  | Xenogenetik | 9354 | breed |
| BW-46 | E. przewalskii | Przewalski horse |  | Zoo Munich | 9355 | prz |
|  |  |  |  |  |  |  |
| **Additional Samples** |  |  |  |  |  |  |
| P090262 | E. caballus | Shetland pony |  | Dworak BGL |  |  |
| BW-44 | E. przewalskii | Przewalski horse |  | Zoo Munich |  |  |
