# Supplementary material for: Identification of Genetic Variation on the Horse Y Chromosome and the Tracing of Male Founder Lineages in Modern Breeds
Source: PLoS One. 2013 Apr 3;8(4):e60015. doi: 10.1371/journal.pone.0060015 (PMC3616054; doi:10.1371/journal.pone.0060015)
Supplement: Table S5 — Primer sequences for Y-SNP verification. (DOCX) [file pone.0060015.s015.docx]

### Table S5. Primer sequences for Y-SNP verification

4 Top candidates and the Przewlaksi SNP in bold. Frequency of the bases in the breed alignment were 86% G and 14% A for Y-25345, 73% T for Y-45288, the minor allele frequency was about 12 % for the region Y-45701/45997. For Y50869 and 27% T was observed in the lipp pool and 85 % in the breed pool.

| **candidate** | **Y-contig/pos** | **amplicon size in bp** | **primer fwd** | | | **primer reverse** | | **mutation** |
| --- | --- | --- | --- | --- | --- | --- | --- | --- |
| **Y-25345** | **YXX_24I23/25345** | **246** | **CCTCCGGCCTTTATGTCTTAG** | | | **TTGGGCTGCAGTATACAACG** | | G/A |
| **Y-45288** | **YE3/10594** | **297** | **CCCTCTGCTGAGCATCTAGG** | | | **TTGGATGAAAGGGACAGTGA** | | T/- |
| **Y-45701/997** | **YE3/1107-11315** | **444** | **CCAACACACGTCAACAGCTC** | | | **GGCTTAGGCCACTGATGGTA** | | 20 SNPs and indels |
| **Y-50869** | **YE17/1277** | **264** | **GGCCTAAGTTGTTCGCAGAG** | | | **TGACTGGTGGTGTCCAGTGT** | | T/A |
|  |  |  |  | | |  |  |  |
| Y-10096 | YXX_24I23 | 369 | GCACACGAGGACTCTGACAA | | | AGTTTGGTCTGTGCCACTCC | | no SNP |
| Y-34616 | YXX_24I23 | 300 | TGGGACTGTTGAAATGGAGA | | | CTTTTCAATGCCATTCCTGA | | no SNP |
| Y-360 | YXX_24I23 | 360 | GATTTGGACCCACCACAGGAG | | | TTGGTTCTCTGGCACCTCTT | | no SNP |
| Y-39624 | YE3 | 290 | GCTGCTCACCAAACACACTG | | | TATGATTCAGCCAGCACCTG | | no SNP |
| Y-40350 | YE3 | 286 | TTCTGAAGAAAGCACCATTTC | | | AAGCGGAGCATGTTAACCAC | | no SNP |
| Y-43717/980 | YE3 | 760 | TGATGGAGAGGTTGTGGAGA | | | AGCTGTGGCATCATGTTCTG | | no SNP |
| Y-68305 | YE3 | 499 | ATCCATGCTTCCTCCCTCTT | | | GCCACAGGGAACTGTGATTT | | no SNP |
| Y-88903 | YM23 | 334 | CCACAGTAACCCCTCCTCAC | | | CAGGGAACCACTAGCCTGAG | | no SNP |
| **Y-90117** | **YM23** | **383** | **GAGCCTTCACCATCTTGTCC** | | | **TAGCTTTCGTCGCAGAAGGT** | | **no SNP, Prz1 G/Prz2 A** |
| Y-94524 | YM23 | 476 | CACAGCTCAGTGGCAGTTGT | | | GCCCTTGCCAGTTATTCTTG | | no SNP |
| Y-95696 | YM23 | 385 | ATGTCTGGGGAGACAGCATC | | | GACCTGCTGTGCTCATCTTG | | no SNP |
| Y-125058 | YNO167 | 469 | AATGGGGTAGGGGATAGGTG | | | CCAATTTCCCCAAGCATTTA | | no SNP |
| Y-151426 | YNO217 | 502 | GAAAGCATGCACTGGCCATATC | | | GCTCCACCCAGAGAAGATGA | | no SNP |
| Y-154256 | YNO217 | 284 | CGAACAAAAGGAGCCACAAT | | | CTGGGAAGAAAGGGGATTTC | | no SNP |
| Y-158936 | YNO217 | 554 | CAAGTCTTTCCAATGGCACA | | | CCCGGAAAAGACTTCAACTGC | | no SNP |
| Y-167086 | YP41 | 382 | ATTCAAGCCCATTGCACATT | | | TAAGAGCCTCCACCAGGATG | | no SNP |
| Y-168785 | YP41 | 531 | TGCCTGTTTTCCAGTTAGGC | | | CCCATTCATGCTCTTCACCT | | no SNP |
|  |  |  |  | | |  |  |  |
| Y-43647/62.fwd | YE3 | 320 | AATGGGCTGGAGACATGAAC | | | CAGTAGTGGGATGGCTGGAT | | not Y spec |
| Y-119468/973.fwd | YNO167 | 413 | CCTGAGATGGAGTCCTGCTG | | | AATCCTCATCATACCTATGCCTTA | | PCR not working |
|  |  |  |  | | |  | |  |
| Ancient domestic variants discovered by Lippold et al. 2011 in the Bronze Age domestic horse | | | |  |  |  | |  |
| Eca SH2-B-17 | BAC-E | 438 | GAGTCCTGCCTTTCCCTCTC | | | GCCTCAGGCCTCTCTGTCTA | | dom C / Prz G |
| Amely-intr2 | contig IV (Paria et al., 2011) | 433 | CTTCACGTTCAAATGTGTGACT | | | CTCAGAAGTGGCATGCAAAG | | dom T / Prz C |
