# Supplementary material for: Identification of Genetic Variation on the Horse Y Chromosome and the Tracing of Male Founder Lineages in Modern Breeds
Source: PLoS One. 2013 Apr 3;8(4):e60015. doi: 10.1371/journal.pone.0060015 (PMC3616054; doi:10.1371/journal.pone.0060015)
Supplement: Table S6 — Microsatellite analysis information. Microsatellite PCR primers, labels and observed alleles in E. caballus (n = 100, 42 different breeds) and E. przewalskii (n = 3). (DOCX) [file pone.0060015.s016.docx]

### Table S6. Microsatellite analysis information

Microsatellite PCR primers and labels and observed alleles in *E. caballus* (n=100, 42 different breeds) and *E. przewalskii* (n=3).

| for details see Wallner et al., 2003 | | E.caballus | E.przwalskii |
| --- | --- | --- | --- |
| Eca.YM2.rev | GTTTGCAGCCAGTACCTACCTT | 120 | 120 |
|  | TET-TGGTTCAGATGGTGTATTTTGTT |  |  |
| Eca.YP9.rev-tail | GTTTCTTAACCCTGGACTTTCTTTTGAA | 223 | 223 |
|  | FAM- AAGCACTGCCTTTTGGAATC |  |  |
| Eca.YE1.rev-tail | GTTTCTTGTGTGTCGTGCCGTGTTTAC | 206 | 206 |
|  | TET-CTTCACTCCCGACCAAGAGA |  |  |
| Eca.YJ10rev.tail | GTTTCTTGCCTCCCACAGCCATAC | 220 | 220 |
|  | HEX-AGTTCCCCTGCACACCT |  |  |
| Eca.YA16rev-tail | GTTTCTTGTAGCAACAAAGTAACAC | 162 | 164/166 |
|  | FAM- TGACTGGAAATTGAAGATG |  |  |
