# Supplementary material for: Identification of Genetic Variation on the Horse Y Chromosome and the Tracing of Male Founder Lineages in Modern Breeds
Source: PLoS One. 2013 Apr 3;8(4):e60015. doi: 10.1371/journal.pone.0060015 (PMC3616054; doi:10.1371/journal.pone.0060015)
Supplement: Table S7 — Haplotype distribution for the breeds under investigation. Data table for the phylogeography in Fig. 3A (breed information, number of samples and observed haplotpyes). (DOCX) [file pone.0060015.s017.docx]

### Table S7. Haplotype distribution for the breeds under investigation

### Data table for the phylogeography in Fig 3A (breed information, number of samples and observed haplotpyes).

| Breed origin | Breed | Number of samples (n) | HT1 | HT2 | HT3 | HT4 | HT5 | HT6 | microsatellite typing (n) |
| --- | --- | --- | --- | --- | --- | --- | --- | --- | --- |
| Central Europe | Lipizzan | 78 | 26 | 52 |  |  |  |  | 16 |
| Central Europe | Haflinger | 34 | 34 |  |  |  |  |  | 1 |
| Central Europe | Austrian Warmblood | 33 | 2 | 15 | 16 |  |  |  | 1 |
| Central Europe | Shagya Arabian | 30 | 13 | 17 |  |  |  |  | 2 |
| Central Europe | Noriker horse | 18 | 18 |  |  |  |  |  | 2 |
| Central Europe | Riding pony | 18 | 6 | 4 | 8 |  |  |  | 2 |
| Central Europe | Hucul | 14 |  | 14 |  |  |  |  | 8 |
| Central Europe | Oldenburger | 10 |  | 7 | 3 |  |  |  | 1 |
| Central Europe | Arabian Partbred | 9 | 4 | 4 | 1 |  |  |  | 2 |
| Central Europe | Kladrubian | 9 | 5 | 4 |  |  |  |  | 4 |
| Central Europe | Knabstrup | 8 | 8 |  |  |  |  |  | 2 |
| Central Europe | Friesian | 7 | 7 |  |  |  |  |  |  |
| Central Europe | Camargue horse | 5 |  |  | 5 |  |  |  |  |
| Central Europe | Tigerpony | 5 | 1 | 1 |  |  |  | 3 | 1 |
| Central Europe | Hanoverian | 4 |  | 1 | 3 |  |  |  |  |
| Central Europe | Holstein | 3 |  |  | 3 |  |  |  |  |
| Central Europe | Baroque Pinto | 2 | 2 |  |  |  |  |  | 1 |
| Central Europe | Bavarian Warmblood | 2 |  | 1 | 1 |  |  |  |  |
| Central Europe | Dutch Warmblood | 2 |  | 2 |  |  |  |  | 1 |
| Central Europe | German classic pony | 2 |  | 2 |  |  |  |  |  |
| Central Europe | Rhinelander horse | 2 |  |  | 2 |  |  |  |  |
| Central Europe | Trakehner | 2 |  | 1 | 1 |  |  |  | 1 |
| Central Europe | Anglo-Arab | 1 | 1 |  |  |  |  |  | 1 |
| Central Europe | Hungarian Warmblood | 1 |  |  | 1 |  |  |  |  |
| Central Europe | Konik | 1 | 1 |  |  |  |  |  |  |
| Central Europe | Sachsen-Anhaltiner | 1 |  | 1 |  |  |  |  |  |
| Central Europe | Wuerttemberg | 1 | 1 |  |  |  |  |  | 1 |
| Iberian Peninsula | Lusitano | 11 | 11 |  |  |  |  |  | 4 |
| Iberian Peninsula | Pura Raza Espanola | 9 | 9 |  |  |  |  |  | 3 |
| British Isles | Shetland pony | 39 | 9 | 1 |  |  |  | 29 | 7 |
| British Isles | Welsh pony | 31 | 12 | 16 | 3 |  |  |  | 1 |
| British Isles | English Thoroughbred | 29 |  | 1 | 28 |  |  |  | 3 |
| British Isles | Connemara pony | 8 | 7 | 1 |  |  |  |  | 2 |
| British Isles | Tinker horse | 8 | 8 |  |  |  |  |  | 2 |
| British Isles | Dartmoor pony | 1 | 1 |  |  |  |  |  | 1 |
| British Isles | Fell pony | 1 | 1 |  |  |  |  |  |  |
| British Isles | New Forest pony | 1 | 1 |  |  |  |  |  | 1 |
| British Isles | Shire horse | 1 | 1 |  |  |  |  |  | 1 |
| Northern Europe | Icelandic horse | 25 | 16 |  |  | 9 |  |  | 5 |
| Northern Europe | Norwegian Fjord horse | 15 |  |  |  |  | 15 |  | 3 |
| Northern Europe | Swedish coldblood trotter | 2 | 2 |  |  |  |  |  | 1 |
| Eastern Europe | Arabian Russian | 48 | 28 | 20 |  |  |  |  | 3 |
| Eastern Europe | Orlov trotter | 2 |  | 2 |  |  |  |  |  |
| Asia | Akhal-Theke | 6 |  | 6 |  |  |  |  |  |
| Asia/Africa | Arabian | 5 | 4 | 1 |  |  |  |  | 2 |
| Africa | Barb | 4 | 4 |  |  |  |  |  | 1 |
| North America | Standardbred | 35 |  | 35 |  |  |  |  | 3 |
| North America | Quarter horse | 18 |  | 9 | 9 |  |  |  | 2 |
| North America | Pinto | 6 | 1 | 1 | 4 |  |  |  | 2 |
| North America | Miniature American | 2 | 1 | 1 |  |  |  |  | 1 |
| North America | American Saddlebred | 1 |  | 1 |  |  |  |  | 1 |
| North America | Appaloosa | 1 |  |  | 1 |  |  |  | 1 |
| North America | Paint | 1 |  | 1 |  |  |  |  | 1 |
| North America | Paso Fino | 1 |  | 1 |  |  |  |  |  |
| South America | Mangalarga Paulista | 1 | 1 |  |  |  |  |  | 1 |
| South America | Mangalarga Marchador | 1 |  | 1 |  |  |  |  | 1 |
|  | Σ | 615 | 246 | 224 | 89 | 9 | 15 | 32 | 100 |
|  | Frequency |  | 40,0 % | 36,4 % | 14,5 % | 1,5 % | 2,4 % | 5,2 % |  |
