# Supplementary material for: Identification of Genetic Variation on the Horse Y Chromosome and the Tracing of Male Founder Lineages in Modern Breeds
Source: PLoS One. 2013 Apr 3;8(4):e60015. doi: 10.1371/journal.pone.0060015 (PMC3616054; doi:10.1371/journal.pone.0060015)
Supplement: Table S8 — Primer sequences for the Sequenom analysis and the screening of the deletions. (DOCX) [file pone.0060015.s018.docx]

### Table S8. Primer sequences for the Sequenom analysis and the screening of the deletions

| **SNP_ID** | **PCR primer 1** | **PCR primer 2** |
| --- | --- | --- |
| Y-E17.1_1277 | ACGTTGGATGGGCTTCCATGAATGACTCTC | ACGTTGGATGCTGCAACCAACAGAAGTGTG |
| Y-E3.1_10594 | ACGTTGGATGGTTGCCAAACTACTCACCAG | ACGTTGGATGATTGTGGGTGAGACTGTGAG |
| Y-E3.1mut_SNP1 | ACGTTGGATGTTGATCTGCAGCGGTAAGAC | ACGTTGGATGTCAGCCTGGGGGCTGGGT |
| Y-E3.1mut_SNP2 | ACGTTGGATGAGTGATGCCCATCCAGATGC | ACGTTGGATGCTTAGTGTAGTGCCTGGTGC |
| YXX_24I23_25345 | ACGTTGGATGGGAATCTACACATCTCACAG | ACGTTGGATGATAGATGGGGAAAAGGTTG |
|  |  |  |
| **SNP_ID** | **Extension primer** | Variants |
| Y-E17.1_1277 | TGACTCTCGAGTTCTACA | T/A |
| Y-E3.1_10594 | TCTAATCAGCATTAGAACAAGT | T/- |
| Y-E3.1mut_SNP1 | AGTTATAGACCATCTCTGGG | T/A |
| Y-E3.1mut_SNP2 | ACCCAAACCCCTCATTG | C/A |
| YXX_24I23_25345 | ACGAAAGACATAAACTACGTTAAA | G/A |

Screening PCR HT6- Shetland pony deletion (966 bp)

| **SNP_ID** | **PCR primer 1** | **PCR primer 2** | **Amplicon HT1-5** | **Amplicon HT6** |
| --- | --- | --- | --- | --- |
| Y_E3.1.11076-12042del | CCCTTTCATCCAACTGCCTA | TATTCCTGGCCAAGTTCTGG | 2010bp | 1044 bp |

Screening for HTPrz- 3051 bp deleted in Przewalski horses

| **SNP_ID** | **PCR primer 1** | **PCR primer 2** | **Amplicon HT1-6** | **Amplicon Prz** |
| --- | --- | --- | --- | --- |
| Y-P16.2241-5412del | GTTCTTCCCAATTTTAACGTGTC | CTTGTTCAGCCTTCGGAATC | 3796 bp | 745 bp |
