# Supplementary material for: Identification of Genetic Variation on the Horse Y Chromosome and the Tracing of Male Founder Lineages in Modern Breeds
Source: PLoS One. 2013 Apr 3;8(4):e60015. doi: 10.1371/journal.pone.0060015 (PMC3616054; doi:10.1371/journal.pone.0060015)

### Table S9. Alignments and Polymorphic sites

The polymorphic position is numbered according position in HT1 in the sequence alignment. Dots indicate identity with the HT1 variant. Comparison of the polymorphic sites within *E.caballus* with the sequence from *E. przewalskii* allows the identification of the ancestral base in HT1 at all positions. Complete sequence alignments are available under accession numbers JX646942 – JX647045


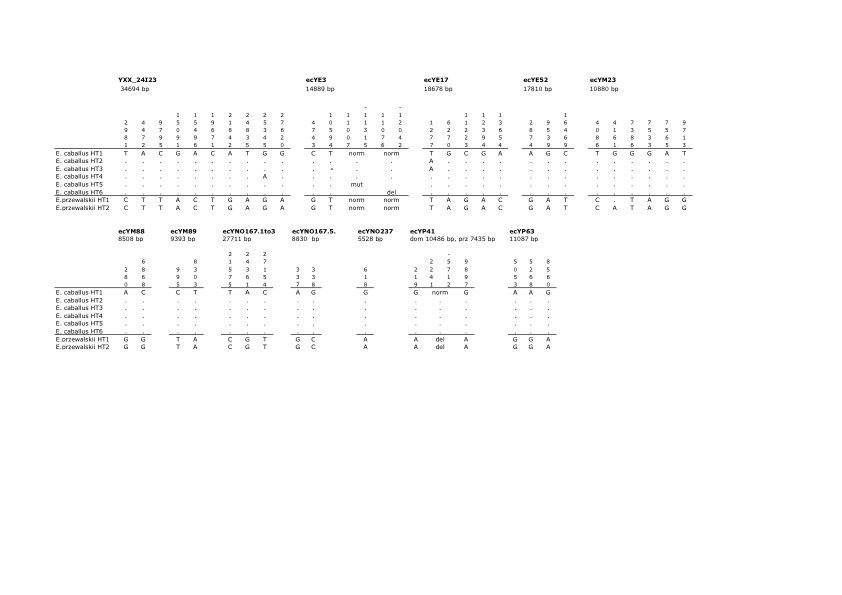

Supplement: Table S9 — Alignments and Polymorphic sites. (DOCX) [file pone.0060015.s019.docx]
