# Supplementary material for: Identification of Genetic Variation on the Horse Y Chromosome and the Tracing of Male Founder Lineages in Modern Breeds
Source: PLoS One. 2013 Apr 3;8(4):e60015. doi: 10.1371/journal.pone.0060015 (PMC3616054; doi:10.1371/journal.pone.0060015)
Supplement: Table S10 — Y-chromosomal dbSNPs. (DOCX) [file pone.0060015.s020.docx]

### Table S10. Y-chromosomal dbSNPs

YXX_24I23-Pos25345, YE17-Pos1277 and YE3-Pos10594 have been submitted to the NCBI dbSNP database (ss#711581504, ss#711581506, ss#711581507)

**YXX_24I23 - Pos 25345 [A/G]**

ATTATAAAATTTTAACAGTATGCGTAAAGTCCATCGTTTGTTTTAACCAGTATTTGCTGTTAAGTAATCACGGTTTAACTTTTTCTCAGTAAAAGCTTATGTTCCCTCCGGCCTTTATGTCTTAGCATTTTAAAAACCTGTGGAAGGATAAAATTAGTACCAAGATAAATGAGGATAGATGGGGAAAAGGTTGAAAATAC[A/G]TTTAACGTAGTTTATGTCTTTCGTAAAACTTAAGACTATTAACTGTGAGATGTGTAGATTCCAGACCATATTTTTTATTGTTTACTTTTAGGTTAAAAAAAACAAGCCATGTCATGTTTTTAAAGTGAACGTTGTATACTGCAGCCCAAAAGAATTATCAGAGGCCTTCCAATCTAACTTTTGCGCAACTTTTTATCAAA

**YE3 - Pos 10594 [T/-]**

TCATTAAAGGCTGACATCATTCTTAACGGCAGCAGTCTACAGTAGGGAGGGTCCCTAATCCATTTCAGTAACTTCCCTCTGCTGAGCATCTAGGATTCCAGAAATGAAATCTGGGTCCAAGATAAGTCTTTTGAAACATGTTGCCAAACTACTCACCAGAAAAGCTGTCAGATTCGTAGTCTAATCAGCATTAGAACAAGT[T/-]AAGTGCTTATTTCTCACAG

TCTCACCCACAATGGGCACTGCTCTTCTATTAGATCTCTGTCAACTTGACAGTAAAACCTAGTGTCTCACTTTTAAACTTGTAGGTCCTAGTTAAACTCTTTTTATATGCTTGTTTCCTATATGTACTTCTCACTGTCCCTTTCATCCAACTGCCTACTGGGGTATAAATTCCTTTCTTT

**YE3 - Pos 1007-12040 see Fig. S5**

**YE17 - Pos 1277 [A/T]**

AGATCAGCAATGGAAAGCATGGCTGGTGTATCTGAGGTGGTGAGAGAATCATACTTGAGAGCACATGTTGCAAAGCCAAGATGCAGGGCCTAAGTTGTTCGCAGAGAGCAAGATTACCTCCTGGGACAACTAGAGCAGGACCCAGAGGTGCTGCCTTTGCAACAGAGCTAGGCTTCCATGAATGACTCTCGAGTTCTACA[A/T]TCACAGTTTGAGATTACTGTTTAGAAAGCACACTTCTGTTGGTTGCAGGCGTGAATAACAGTGATTGTATAAAGTGTAGAATCACTTATTAGAAATCAGAGACAATCTATCTGTTGTTCAGCCGGGTGGACACTGGACACCACCAGTCAGGCTGTCCGATGCTGGAGAACAACGATGCTACCAAGCCTTAGGACAGGGGC

**YM23 - Pos 4161 [G/A]**

AACTCAGTCTTCCCTGCTAGCCCTTGGGGTTCTGAGCTTGCTCCCTCAGAAGAGATAGGACCTTGCCTTTAGGTTTCCCGCTCCCGCCTGCTAGACCCGCCGGTGCCCAGCCCCCGGAGTCCTGGTGAATGAGCTACAGAGGCTCCAGGGAATTGAACTGCGGCGAAAGCAGGTACACGTCCCACGTGGAACACAGATTT[G/A]CAGACCTTCTGCGACGAAAGCTATGAGGCACACGCCACATAGCCAAAAATTGCTGCCCTCCAGGAAAAATGAAATGCAGAAGAATAGCTTTCAAGGCTGGGATTAGGAGCCAAAGCAGATACTCCAATTGAAACTGCGGATGAAGCAGTCAAAATTTCCCTAGATTGGGGTGAAGCTGTGCCTTGTACGCACAAGATTAA
