# Supplementary material for: Identification of Genetic Variation on the Horse Y Chromosome and the Tracing of Male Founder Lineages in Modern Breeds
Source: PLoS One. 2013 Apr 3;8(4):e60015. doi: 10.1371/journal.pone.0060015 (PMC3616054; doi:10.1371/journal.pone.0060015)
Supplement: Table S11 — Y-chromosomal haplotypes. (DOCX) [file pone.0060015.s021.docx]

### Table S11. Y-chromosomal haplotypes

The polymorphic postions are numbered according to their location on the LRP contig in HT1 (contig identifiers are listed in Table S3)

| **Locus** | **YXX_24I23** | **YE3** | **YE3** | **YE17** | **YE17** | **YM23** |  |  |
| --- | --- | --- | --- | --- | --- | --- | --- | --- |
| **Position** | 25345 | 10594 | 11007 -11315 | 11076-12042 | 1277 | 4161 |  |  |
| **HT1** | G | T | normal | normal | T | G |  |  |
| **HT2** | G | T | normal | normal | A | G |  |  |
| **HT3** | G | - | normal | normal | A | G |  |  |
| **HT4** | A | T | normal | normal | T | G |  |  |
| **HT5** | G | T | mutated |  | T | G |  |  |
| **HT6** | G | T |  | deleted | T | G |  |  |
| **HTprz1** | G | T | normal | normal | T | G | 37 SNPs; 3051 bp deletion | |
| **HTprz2** | G | T | normal | normal | T | A | 38 SNPs; 3051 bb deletion | |
